# A multimodal approach to depression diagnosis: insights from machine learning algorithm development in primary care

**DOI:** 10.1007/s00406-025-01990-5

**Published:** 2025-03-10

**Authors:** Julia Eder, Mark Sen Dong, Melanie Wöhler, Maria S. Simon, Catherine Glocker, Lisa Pfeiffer, Richard Gaus, Johannes Wolf, Kadir Mestan, Helmut Krcmar, Nikolaos Koutsouleris, Antonius Schneider, Jochen Gensichen, Richard Musil, Peter Falkai

**Affiliations:** 1 Department of Psychiatry and Psychotherapy, LMU University Hospital, LMU Munich, Nussbaumstraße 7, 80336 Munich, Germany; 2 Graduate Program “POKAL - Predictors and Outcomes in Primary Care” (DFG-GrK 2621), Munich, Germany; 3 German Center for Mental Health (DZPG), Partner Site Munich-Augsburg, Munich, Germany; 4 Chair for Information Systems, Technical University of Munich (TUM), 85748 Garching, Germany; 5 Institute of Psychiatry, Psychology and Neuroscience, King’s College London, London, UK; 6 Institute of General Practice and Health Services Research, School of Medicine, Technical University Munich, Munich, Germany; 7 Institute of General Practice and Family Medicine, Ludwig-Maximilians-University Munich, Munich, Germany; 8 Oberberg Fachklinik Bad Tölz, Bad Tölz, Germany; 9 Max-Planck Institute of Psychiatry, Munich, Germany

**Keywords:** Depression diagnosis, Primary care, Depression subtypes, Data-driven methods, Predictive algorithm

## Abstract

General practitioners play an essential role in identifying depression and are often the first point of contact for patients. Current diagnostic tools, such as the Patient Health Questionnaire-9, provide initial screening but might lead to false positives. To address this, we developed a two-step machine learning model called *Clinical 15*, trained on a cohort of 581 participants using a nested cross-validation framework. The model integrates self-reported data from validated questionnaires within a study sample of patients presenting to general practitioners. *Clinical 15* demonstrated a balanced accuracy of 88.2% and incorporates a traffic light system: green for healthy, red for depression, and yellow for uncertain cases. Gaussian mixture model clustering identified four depression subtypes, including an Immuno-Metabolic cluster characterized by obesity, low-grade inflammation, autonomic nervous system dysregulation, and reduced physical activity. The *Clinical 15* algorithm identified all patients within the immuno-metabolic cluster as depressed, although 22.2% (30.8% across the whole dataset) were categorized as uncertain, leading to a yellow traffic light. The biological characterization of patients and monitoring of their clinical course may be used for differential risk stratification in the future. In conclusion, the *Clinical 15* model provides a highly sensitive and specific tool to support GPs in diagnosing depression. Future algorithm improvements may integrate further biological markers and longitudinal data. The tool’s clinical utility needs further evaluation through a randomized controlled trial, which is currently being planned. Additionally, assessing whether GPs actively integrate the algorithm’s predictions into their diagnostic and treatment decisions will be critical for its practical adoption.

**Supplementary Information:**

The online version contains supplementary material available at 10.1007/s00406-025-01990-5.

## Introduction

General practitioners (GPs) encounter many healthy individuals while identifying those with medical conditions that need further attention during their daily practice. Broad medical knowledge is essential for their role [1]. Despite the high prevalence of psychiatric disorders in the general population, GPs in Germany, but also in other countries, are not required to undergo psychiatric training during their subspecialty training [2]. However, it is important to note that all GPs in Germany need to complete 80 h of training in psychosomatic basic care as part of their subspecialty training [3]. GPs accurately diagnose depression in 47.3% of cases and document it in 33.6%, while they are effective at ruling out depression in individuals without the condition (specificity of 81.3%) [4]. Compared to single evaluations, the diagnostic accuracy improves with extended prospective assessments over 3–12 months. These findings underscore the need for tools to enhance GPs’ sensitivity and specificity, reducing misdiagnoses and ensuring timely, accurate detection of depression [4]. In 2023, the United States Preventive Services Task Force recommended universal screening for major depressive disorder in all adults and anxiety disorders in individuals aged 19–64. This approach involves administering symptom questionnaires to patients without psychiatric diagnoses, categorizing the results, conducting thorough assessments for those with positive findings, and implementing necessary treatments [5]. One recommended tool is the Patient Health Questionnaire-9 (PHQ-9)[5].

This is one of the most widely used tools for screening depression in primary care. While it has demonstrated reasonable sensitivity and specificity, the PHQ-9 cannot confirm a clinical diagnosis of depression. Studies suggest that it functions effectively as an initial screening step, with the PHQ-2 often used as a brief alternative due to its ease of use and acceptability. However, in some cases, the PHQ-9 has been shown to perform less accurately than GPs [6, 7].

Despite these efforts, screening for depression in primary care continues to face challenges. Some researchers are concerned that broad screening might lead to a high rate of false positives, leading to the misclassification of healthy individuals as depressed [8, 9]. GPs, often the first point of contact for affected individuals, find diagnosing depression particularly challenging. GPs frequently perceive depression as a “gray area” with symptoms spanning emotional, somatic, and associated dimensions [10]. Malhi et al. emphasize that GPs often consider somatic symptoms as the most crucial predictor regarding depression severity; further misconceptions are frequently observed—for instance, the assumption that the risk of self-harm is synonymous with suicidal intent. [11].

Further complicating this endeavor is the fact that depression is a highly heterogeneous disorder, and diagnosis relies solely on clinical interviews. Research has yet to identify a reliable biomarker to facilitate diagnostic processes.

Since the twentieth century, researchers have pursued biomarkers for depression, beginning with cortisol, but results have been inconclusive [12]. Patients with slightly elevated but traditionally non-pathological CRP levels and metabolic abnormalities are classified as immunometabolic depression (IMD). This is an important distinction because this cohort exhibits worse outcomes than patients without these changes [13]. Penninx et al. identified a correlation between metabolic and immunological dysregulation and atypical depressive symptoms characterized by hypersomnia, increased appetite, weight gain, and mood reactivity. In contrast, melancholic depression is marked by anhedonia, non-reactive mood, psychomotor disturbances, sleep and appetite loss, weight loss, diurnal mood variation, and cognitive impairments. Unlike atypical depression, melancholic depression is more frequently associated with hypercortisolism [14]. The presence of multiple biological subtypes of the same classificational category complicates the identification of specific biological mechanisms that may be causally involved. Since they can dilute involved effects, research in this area is more challenging [15].

This complex diagnostic landscape underscores the urgent need for improved tools and strategies to support GPs in diagnosing depression. These tools must address the nuanced presentation of depressive symptoms in primary care, reduce false positives, and ensure that patients receive timely and appropriate care. Further, there is so far no data on potential subtypes of depression that GPs might encounter [16].

By analyzing data collected in primary care settings, we aim to present a machine learning (ML) prediction model designed to simplify the diagnostic process for GPs. In addition, we investigate depression subtypes using data-driven methods to identify patterns and presentations specific to this context.

## Methods and design

Although this study was designed as a prospective observational study, the current analysis is limited to the cross-sectional data collected at study inclusion. It is a subproject of the research network POKAL (Predictors and Clinical Outcomes in Depressive Disorders; GRK 2621), which is sponsored by the German Research Foundation (DFG) [16]. Detailed descriptions of the study design, eligibility criteria, and assessed metrics have been published elsewhere [17].

In addition to clinical assessments, cardiovascular parameters, metabolic factors, and inflammatory parameters were collected. All participants provided written informed consent before enrollment. The study received approval from the local ethics committee (project nr. 22-0637, date 14.09.2022) and was registered at ClinicalTrials.gov (NCT05547711) and the German Clinical Trials Register (DRKS00030203) [17].

This study cohort comprised patients presenting to general practitioners (GPs) for various health concerns, outpatients from the LMU psychiatric clinic, and volunteers. The LMU research team coordinates and manages recruitment, which is still ongoing.

A subset of 581 participants (of initially 730, incl. 18 dropouts) was included in the analysis, as they had completed the SCID-5 diagnostic assessment at baseline and returned the questionnaires that were part of the assessment. The SCID-5 diagnosis was utilized as the primary target variable for training and evaluating the models. Of these participants, 90 (15.5%) were diagnosed with depressive disorders, while 491 (84.5%) were classified as non-depressed. Of the remaining 581 participants, 28.4% were recruited at the LMU site, and 71.6% came from various GP offices.

### Data overview

The dataset included features categorized into four modalities:

1. **Self-reported measures:** Questionnaires, including the World Health Organization-5 Well-Being Index (WHO-5) [18], PHQ-9[19], World Health Organization Disability Assessment Schedule 2.0 12-items Score (WHODAS 2.0)[20], University of California Los Angeles 3-Item Loneliness Scale (UCLA 3-items), Primary Care Post-Traumatic Stress Disorder Screen (PC-PTSD), Lubben Social Network Scale (LSNS), and the Patient Health Questionnaire-15 (PHQ-15)
2. **Lab-based measures:** Blood test values assessing blood biomarkers
3. **Physiological measures:** Heart rate variability (HRV) indices and bioimpedance scale measurements
4. **Anthropometric data:** Body measurements, such as BMI and waist-to-hip ratios, as well as blood pressure, Pulse, and body temperature
5. **Observer ratings:** Structured Clinical Interview for DSM-5 Disorders (SKID)[21] and the Montgomery-Åsberg Depression Rating Scale (MADRS).

### Statistical analysis

Descriptive analyses were conducted using mean (M) and standard deviation (SD) for continuous variables and frequencies (n) with percentages (%) for categorical variables. Group comparisons between individuals with and without depression were performed using chi-square tests for categorical variables and independent t-tests for continuous variables. Mann–Whitney U tests, or Kruskal Wallis Test were calculated for non-normally distributed data. A p-value < 0.05 was otherwise deemed significant.

### Model development

The supplements (S1) describe the feature engineering steps in further detail. Features representing less than 5% of the population were aggregated into broader categories to prevent overfitting. All models were trained and evaluated using nested cross-validation with five randomly split folds at the outer and inner loops. To estimate the model fit on unseen training sets drawn from the same population [22]. For each outer CV fold, missing values were imputed using the KNN algorithm (n_neighbors = 7), followed by the respective pipelines described in the next section. To account for class imbalance, sample weights were adjusted to ensure a balanced contribution of each class.

#### Pipelines

Four distinct pipelines were implemented to train and optimize the respective models, tailored to the characteristics of the data and project requirements. These pipelines were selected by an expert with domain-specific knowledge, ensuring that the most suitable approach was applied to each data modality. This expert-driven selection process accounted for factors such as data structure, complexity, and the analysis’s goals, maximizing the models’ performance and relevance. These pipelines are described as follows:

#### Pipeline 1: XGBoost

The first pipeline employed XGBoost (eXtreme Gradient Boosting) [23], a scalable, distributed gradient-boosting decision tree library widely used for regression, classification, and ranking problems. This pipeline did not include any preprocessing or feature selection steps, making it suitable for datasets with few features (N < 10). Due to the limited number of features, additional feature selection methods were deemed unnecessary.

#### Pipeline 2: Boruta + XGBoost

Boruta feature selection [24] was integrated into the second pipeline before model training with XGBoost. This approach was designed for datasets with a moderate number of features, where a coarse feature selection step was required to reduce the feature set. This pipeline aimed to improve model efficiency and interpretability by eliminating irrelevant or weakly predictive features.

#### Pipeline 3: Boruta + Greedy Search + XGBoost

The third pipeline combined Boruta feature selection with a greedy forward stepwise wrapper to refine the feature set in two stages. First, Boruta was applied to eliminate non-informative features. The reduced feature set underwent greedy forward stepwise selection to identify the most predictive feature combination. While this method occasionally resulted in slightly lower balanced accuracy (BAC) compared to Pipeline 2, it used significantly fewer features, enhancing the model’s practical applicability in clinical environments.

#### Pipeline 4: PCA + SVM

The fourth pipeline utilized Principal Component Analysis [25] to transform the original features into principal components, capturing the dominant signal from highly correlated features. These components were subsequently used to train a Support Vector Machine with a linear kernel [26]. This pipeline was particularly suitable for datasets with high inter-feature correlation, such as those derived from blood test values and heart rate variability measurements.

#### Model training and optimization

Based on the five data modalities, we trained five base models: one based on biographic data and social network size, a second based on somatic problems reported on the PHQ-15, a further model combining the PHQ-9 and the WHO-5 questionnaire—self-rating concerning depression symptomatology, a model on loneliness, trauma, PHQ9, WHODAS, and PHQ-15, and a fifth model combining all questionnaires with the biographic data (see Table 2).

Further, stacked models were trained. These models use individual prediction scores from base models as input to train a hierarchical optimizer. Specifically, the StackingClassifier Class form scikit-learn was used with a logistic regression function as the final estimator [27].

Additionally, we trained a sequential model. This model combines different base and stacker models in a stepwise manner optimized for prediction performance and reduces the per-case examinations needed to achieve this performance, which is an innovative method to maximize prediction accuracy by using multiple modalities while reducing the additional burden and cost for acquiring more data. The sequence optimizer aims to explore a range of propagation thresholds, strategically selecting individuals whose diagnostic accuracy would benefit from integrating additional modalities into the stack [28]. The propagation thresholds were also utilized to create a “traffic light” risk stratification system, categorizing levels of certainty as follows: low certainty (50–66%), medium certainty (67–82%), and high certainty (83–100%). If there is a certainty above 67% that an individual suffers or does not suffer from depression, the traffic light is either green or red; in any other case, the light is yellow. The results were also compared with the PHQ-9 at a cutoff of > 8 [29]. Python 3.6.13 was used for model development with pandas (version 1.1.5) and xgboost (version 0.90). Feature selection was performed using the Boruta library (version 0.3).

#### Gaussian mixture Model

To cluster the depressed cohort (n = 90), we used the Gaussian mixture model (GMM) on the laboratory assessments, the HRV data, and the anthropometric data. No questionnaire data was included.

As the data was not normally distributed, we applied a Yeo-Johnson transformation before clustering. Non-normality was graphically assessed using Q–Q plots before and after the transformation (see Supplements S6). The optimal number of clusters was determined by evaluating AIC, BIC, and silhouette scores, leading to the selection of four clusters with a full covariance structure. Based on the imputed but not transformed data, the clusters were compared using chi-square tests for categorical variables and either Kruskal–Wallis tests or one-way ANOVA for continuous variables, depending on the presence of a normal distribution. In cases of significant results, appropriate post hoc tests were performed: pairwise chi-square tests with Bonferroni correction for categorical variables, Dunn’s post hoc tests for non-normally distributed continuous variables, or Tukey’s HSD test for normally distributed continuous variables.

GMM is a data-driven method; based on BIC and AIC metrics, four clusters were chosen (S7). These clusters were named post hoc according to their characteristics. The first cluster was called the *Adaptive cluster*, as it showed normal values with only a non-significant cortisol elevation, suggesting an ongoing physiological adaptation to depression or external stressors. One cluster, comprising metabolically healthy elderly individuals, was named *Late Depression*. The third cluster, named *Immuno-Metabolic*, demonstrated autonomic nervous system dysregulation, low-grade inflammation, lower IGF-1 levels, and an unfavorable metabolic profile. The fourth cluster, called the *Overweight, Non-Inflammatory* cluster, was characterized by abnormal blood lipid levels, suboptimal body composition, lower CRP levels, and no ANS dysregulation.

## Results

The study cohort consisted of outpatient individuals with (n = 90) and without (n = 491) depression, as determined via a SCID-5 rating. Among the entire cohort, 271 participants reported having sought professional psychiatric or psychological help in the past, with a mean age of 29.57 years (SD = 13.38) at the time of their first admission. Additionally, 16.5% of participants reported that they were currently taking psychiatric medication.

The cohort included male and female participants, with a majority being female, 61.5% in the whole dataset. The mean age of the overall cohort was 42.98 years (SD = 14.48). Among non-depressed individuals, the mean age was slightly higher at 43.47 years (SD = 14.68), while depressed individuals had a lower mean age of 40.34 years (SD = 13.10, t(570) = 3.79, p = 0.052). Educational attainment was categorized as low, medium, or high. Low, medium, and high education levels were observed in 5.5%, 17.9%, and 74.9% of all individuals. In the non-depressed group, these rates were 5.4%, 17.3%, and 77.3%, while in the depressed group, they were 6.7%, 23.3%, and 70.0% (p = 0.21). Depressed individuals reported financial difficulties within the last 12 months more frequently (23.3% multiple times, 11.1% once, 63.3% never) than non-depressed individuals (4.3% multiple times, 5.7% once, 88.4% never; p < 0.001). The overall mean working hours per week was 31.55 (SD = 12.72). Among depressed individuals, the mean was 28.95 (SD = 13.74, N = 81), and for non-depressed individuals, it was 32.05 (SD = 12.47; H(1, N = 502) = 7.64, p = 0.05). 54.4% reported smoking daily (n = 311), 30.1% had quit smoking (n = 172), and 15.6% had never smoked (n = 101). There were no significant differences between depressed and not depressed individuals (X^2^(2) = 0.46, p = 0.80) in terms of their smoking habits.

Overall, 63.6% of participants were currently in a partnership. Among participants without depression, 67.2% were in a partnership, compared to 44.4% of participants with depression (X^2^(1) = 16.91, p =  < 0.001). Further details concerning depressed and not depressed individuals, as well as the whole dataset, can be seen in Table 1.

**Table 1:** Descriptive statistics and group comparisons for depressed and non-depressed groups

Variable	Non-Depressed (N = 491) M (SD)	Depressed (N = 90) M (SD)	p-value
Age (years)	43.47 (14.68)	40.34 (13.10)	0.052
Sex (%, female)	60.1%	68.9%	0.14
Binge Drinking (%, yes)	60.2%	50.0%	0.15
Depressive episode before recent episode (%, yes)	10.6%	71.1%	< 0.001***
Currently Taking Psychiatric Medication (%, yes)	8.4%	59.6%	< 0.001***
Number of Depressive Episodes Over Lifetime	0.30 (1.39)	5.90 (9.79)	< 0.001***
Total Body Weight (kg)	73.58 (17.48)	72.67 (17.29)	0.53
Total Muscle Mass (kg)	52.26 (10.60)	49.57 (9.55)	0.04
Total Body Water (kg)	37.17 (8.26)	35.29 (7.42)	0.06
Body Mass Index (BMI, kg/m^2^)	24.42 (4.91)	24.49 (5.05)	0.76
Body Fat Percentage (%)	24.39 (7.67)	26.50 (8.57)	0.06
Visceral Fat Score	5.73 (4.64)	5.29 (4.35)	0.35
Basal Metabolic Rate (BMR, kcal)	1631.55 (324.97)	1670.96 (706.66)	0.23
Waist-to-hip ratio	0.85 (0.10)	0.83 (0.10)	0.29
Waist circumference (cm)	81.70 (14.60)	81.10 (14.23)	0.97
Hip circumference (cm)	96.47 (11.14)	98.66 (13.63)	0.15
Body temperature (°C)	36.43 (0.44)	36.51 (0.45)	0.07
Resting Heart rate (bpm)	70.78 (12.45)	75.79 (13.48)	< 0.001***
Diastolic blood pressure (mmHg)	80.47 (10.41)	81.87 (10.25)	0.13
Systolic blood pressure (mmHg)	124.96 (17.39)	124.70 (17.14)	0.96
Heart Rate (bpm), Lying Position	68.05 (9.81)	69.55 (9.79)	0.13
HRV (SDNN, ms), Lying Position	45.53 (24.56)	39.64 (20.16)	0.10
HRV (RMSSD, ms), Lying Position	43.17 (31.01)	37.76 (24.30)	0.26
HRV (LF, ms2), Lying Position	1351.14 (1452.71)	990.16 (1022.93)	0.02*
HRV (HF, ms2), Lying Position	972.55 (1380.75)	799.09 (1077.74)	0.36
HRV (Baevskii Index) Lying Position	170.95 *(*247.96)	224.24 (271.75)	0.03*
Glucose level (mg/dL)	75.41 (14.06)	80.06 (17.60)	0.001**
CRP (mg/dL)	0.23 (0.70)	0.28 (0.51)	0.29
AST (U/L)	23.97 (7.76)	22.99 (8.73)	0.10
ALT (U/L)	23.36 (13.08)	23.50 (12.48)	0.89
Gamma-GT (U/L)	20.86 (19.87)	22.14 (27.16)	0.88
Total cholesterol (mg/dL)	192.32 (40.60)	201.01 (42.93)	0.09
Triglycerides (mg/dL)	93.71 (52.77)	108.19 (57.55)	0.02*
LDL-cholesterol (mg/dL)	114.64 (34.68)	122.36 (38.52)	0.10
HDL-cholesterol (mg/dL)	61.76 (15.87)	61.78 (13.81)	0.73
Non-HDL Cholesterol (mg/L)	132.53 (39.82)	135.22 (44.39)	0.05
HbA1c (NGSP, %)	5.29 (0.53)	5.29 (0.46)	0.53
Leukocyte count (× 10⁹/L)	5.49 (1.50)	6.15 (1.79)	0.002**
Erythrocyte Count (10^12^/L)	4.72 (0.44)	4.71 (0.39)	0.83
Hemoglobin (g/dl)	14.01 (1.59)	13.98 (1.33)	0.66
Hematocrit (%)	41.35 (3.48)	41.19 (3.35)	0.70
Platelet count (× 10⁹/L)	251.03 (62.11)	271.39 (68.66)	0.01*
Neutrophil count (× 10⁹/L)	3.09 (1.14)	3.55 (1.26)	< 0.001***
Monocyte count (× 10⁹/L)	0.43 (0.13)	0.48 (0.17)	0.03*
Eosinophil Count (10^9^/L)	0.16 (0.15)	0.17 (0.17)	0.92
Basophil count (× 10⁹/L)	0.04 (0.02)	0.05 (0.02)	0.02*
Normoblast Count (10^9^/L)	0.10 (0.00)	0.10 (0.02)	0.35
Lymphocyte Count (10^9^/L)	1.75 (0.54)	1.88 (0.59)	0.07
Monocyte Count (10^9^/L)	0.43 (0.13)	0.48 (0.17)	0.03
TSH (µU/ml)	2.03 (1.43)	1.93 (1.04)	0.90
Cortisol (µg/dL)	13.88 (7.03)	14.07 (6.67)	0.71
IGF-1 (ng/mL)	163.31 (59.65)	160.87 (56.05)	0.83
Alpha-1-antitrypsin (g/L)	1.36 (0.22)	1.41 (0.22)	0.049
MADRS score	4.72 (4.93)	22.30 (8.03)	< 0.001***
WHODAS 2.0 score	7.46 (8.09)	24.01 (10.05)	< 0.001***
WHO-5 score	18.15 (4.84)	11.23 (4.11)	< 0.001***
PHQ-9 score	4.77 (3.51)	14.68 (5.18)	< 0.001***
UCLA 3-items score	5.27 (1.78)	7.69 *(*2.34)	< 0.001***
PC-PTSD score	0.57 (1.10)	1.98 (1.84)	< 0.001***
Lubben social network scale	14.35 (4.75)	13.58 (6.80)	< 0.02*
GAD-7 Score	4.20 (3.74)	11.59 (5.21)	< 0.001***
PHQ-15 score	5.82 (3.84)	11.64 (5.34)	< 0.001***
Number of siblings	1.30 ( 0.86)	1.39, (0.94)	0.40
Adults in household	1.93 (0.74)	1.88, (0.93)	0.17
Partner from another ethnicity (%, yes)	21.92%	18.51%	0.70
Separation in the last 12 months (%, yes)	6.70%	15.9%	0.01*
Sport ≥ 1 × per week (%, yes)	77.26%	54.44%	< 0.001***
Heart attack (%, yes)	5.92%	3.41%	0.49
Employment in the last 12 months (%, yes)	86.24%	82.8%	0.49
Biological children (%, yes)	44.12%	34.1%	0.10
Migration background (%, yes)	23.44%	25.84%	0.72
Caring for relatives (%, yes)	9.3%	4.6%	0.21
Moved during childhood/adolescence (%, yes)	60.00%	68.2%	0.19
Born in Germany (%, yes)	87.0%	89.3%	0.69
Mother tongue German (%, yes)	90.11%	90.5%	0.99
Parents separated (%, yes)	26.13%	44.94%	< 0.001***
Attempted suicide (%, yes)	2.51%	20.22%	< 0.001***

*p < 0.05, **p < 0.01, ***p < 0.001

*MADRS* Montgomery–Åsberg Depression Rating Scale, measuring the severity of depressive symptoms, *WHODAS 2.0 Score* World Health Organization Disability Assessment Schedule 2.0 12-item score, *WHO-5 Score* World Health Organization-5 well-being index, *PHQ-9 Score* Patient Health Questionnaire-9, *UCLA 3-items Score* UCLA Loneliness Scale, 3-item version, *PC-PTSD Score* Primary Care PTSD Screen, *GAD-7* Generalized Anxiety Disorder 7-item, *PHQ-15 Score*: Patient Health Questionnaire 15, *IGF-1* Insulin-like Growth Factor 1, *CRP* C-reactive protein, *TSH* thyroid stimulating hormone, *AST* aspartate aminotransferase, *ALT* alanine aminotransferase, *LDL* low-densitiy lipoprotein, *HDL* high-densitiy lipoprotein cholesterol, *gamma-GT* gamma-glutamyl transferase

Initially, we calculated base models by applying a single machine learning algorithm, typically XGBoost, within a nested cross-validation (CV) framework, depending on the pipeline (see Methods and Design: Model Development).

Compared to the baseline models based on self-rating questionnaires, none of the anthropometric base models achieved a BAC above 60 (see Table 2). Among the physiological measures, the HRV-based model demonstrated the best performance, while the model relying solely on somatic measures, such as vital parameters and waist-to-hip ratio, performed the worst.

**Table 2:** Cross-validation performance of base models using biomarker and self-rated features

Model name	Model type	Modalities	Features	Pipeline	BAC	Sens	Spec
Somatic metrics	Base	Waist-to-hip ratio, blood pressure, temperature	9	P1	56.0	46.7	65.4
HRV	Base	Heart rate variability measures	26	P4	59.8	55.6	63.9
Lab	Base	Blood test values	36	P4	57.4	41.1	73.7
Biographic	Base	Biographical data + LSNS-6	18	P2	74.2	67.8	80.7
somatic	Base	PHQ15	5	P3	78.5	78.9	78.0
Depression items	Base	WHO-5 + PHQ-9	11	P3	86.3	84.4	88.2
All clinical	Base	WHO-5 + UCLA + PHQ-9 + WHODAS2.0 + PC-PTSD + PHQ-15	19	P3	85.3	82.2	88.4
All self-rated	Base	All clinical + biographic + LSNS	16	P3	87.8	87.8	87.8
Clinical 15	Base	WHO-5 + PHQ-9 + WHODAS2.0	15	P1	**88.2**	**87.8**	**88.6**

The best performing model is marked bold

Performance metrics are derived from nested cross-validation

*BAC* balanced accuracy; *Sens* sensitivity; *Spec* specificity, *HRV* heart rate variability, *PHQ-15* Patient Health Questionnaire-15, *WHO-5* World Health Organization Well-Being Index, *PHQ-9* Patient Health Questionnaire-9, *WHODAS 2.0* World Health Organization Disability Assessment Schedule 2.0, *PC-PTSD* Primary Care Post-Traumatic Stress Disorder Screen, *UCLA* 3-item Loneliness Scale, *LSNS-6* Lubben Social Network Scale-6

A model we called *Clinical 15* yielded the best overall performance. It consisted of 15 items derived from the PHQ-9, the WHO-5, and the WHODAS 2.0. The performances of the base models can be seen in Table 2. The feature importances of the base models can be found in the Supplements (S2).

The stacked models combined predictions from all base models and did not outperform the best individual model. Specifically, the self-rated model, integrating subjective clinical inputs, consistently yielded superior performance metrics compared to any stacked approach. The respective data can be found in the supplements (S3).

The sequential model consisted of different features from the WHODAS 2.0, PHQ9, and WHO 5 questionnaires. The notion of the sequential model was further to improve the usability of the *Clinical 15* model. We split the first five items with the greatest feature importance into a first step and the remaining 10 items into a second half. Notably, the *Clinical 5* (first step) model reduced the need for comprehensive evaluation by identifying that only 25% of individuals required further assessment beyond the initial feature set. The sequential framework demonstrated strong classification performance, achieving a mean BAC of 88.4% within the nested-cross validation framework. This was accompanied by a mean sensitivity of 87.8% and a mean specificity of 89.0%. If considering the traffic light system (See Methods and Design: Model Training and Optimisation), the sensitivity for depression was 93.2%, and the corresponding specificity was 93.3% (see Table 3). Using the PHQ-9 with the cutoff of > 8, as proposed by Levis et al. [29] and recommended by the US Preventive Services Task Force, we calculated the classification metrics for the PHQ-9 within our sample. At this cutoff, we achieved a balanced accuracy of 83.7%. This analysis was conducted to compare the performance of this established tool with our algorithm.

**Table 3:** The performance of sequential model depicting the traffic light systems and the probabilities of correct and incorrect disease classification

Model name	Total		High certainty groups		Low certainty groups	
			Red traffic light	Green traffic light	Yellow traffic light	
	Depression	HC	Depression	HC	Depression	HC
Clinical 5 (first step)	76/90 (84.4%)	424/491 (86.4%)	38/45 (84.4%)	219/235 (93.2%)	38/45 (84.4%)	205/256 (80.1%)
Clinical 15 (including second step)	**79/90 (87.8%)**	**436/491 (88.8%)**	**55/59 (93.2%)**	**320/343 (93.3%)**	24/31 (77.4%)	116/148 (78.4%)

The best performing model is marked bold

The table shows the number of correctly classified instances versus the total number of true instances for both classes in the clinical sequential system

*HC* healthy control

Adding biological markers like HRV and blood biomarkers after the first two steps did not increase the BAC and was deemed unnecessary for the diagnostic algorithm with the current training data.

GMM clustering identified four distinct clusters, including an *Immuno-Metabolic* cluster (n = 9) that also showed autonomic nervous system (ANS) dysregulation as determined by a five-minute HRV measurement (see Table 4). Another cluster, which we named Overweight, Non-Inflammatory (n = 28), exhibited abnormal blood lipid levels and suboptimal body composition but lower CRP levels. Also, the dysregulation of the ANS, as determined by a five-minute HRV measurement, was distinct for the Immuno-Metabolic subtype. All nine individuals assigned to the *Immuno-Metabolic* cluster were correctly identified as depressed by the *Clinical 15*. Two individuals were flagged as uncertain, leading to a yellow and not a red color coding, but they were still correctly diagnosed. In the *Overweight, Non-Inflammatory* cluster, 3 out of 28 individuals were incorrectly classified, all associated with a yellow traffic light, as the sequential algorithm was not certain concerning its prediction. In the *Adaptive* cluster (4/26) and in the *Late Depression* cluster (4/27), four participants were misclassified in both groups, wherein in each group, two were in the low certainty group, while it was confident about two incorrect predictions.

**Table 4:** Shows descriptive statistics as well as group comparisons of clusters that were found through GMM

	Late depression, n = 27, M (SD)	Adaptive, n = 26, M (SD)	Overweight non-inflammatory, n = 28, M (SD)	Immuno-metabolic, n = 9, M (SD)	p-value
Age at Study Inclusion (years)	50.76 (10.85)	33.20 (9.97)	35.39 (11.05)	45.27 (11.77)	< 0.001
Sex (%, female)	88.9%	80.8%	42.9%	55.56%	0.001
Binge drinking (%, yes)	18.5%	49.5%	65.8%	52.4%	0.001**
Depressive episode before recent episode (%, yes)	70.4%	69.23%	82.14%	44.44%	0.20
Currently taking psychiatric medication (%, yes)	70.4%	40.7%	64.3%	66.67%	0.12
Number of depressive episodes over lifetime	4.23 (4.11)	4.23 (3.47)	5.30 (8.87)	9.63 (15.32)	0.35
Total body weight (kg)	67.76 (9.45)	62.41 (8.01)	80.33 (12.89)	94.91 (28.89)	< 0.001
Total muscle mass (kg)	44.31 (5.22)	46.05 (5.95)	55.86 (8.42)	57.44 (11.93)	< 0.001
Total body water (kg)	31.48 (3.38)	31.89 (3.94)	40.19 (6.15)	42.42 (10.52)	< 0.001
Body mass index (BMI, kg/m^2^)	24.01 (3.30)	21.57 (2.37)	25.38 (3.92)	31.51 (8.45)	< 0.001
Body fat percentage (%)	28.70 (7.58)	22.07 (5.14)	26.14 (8.60)	33.84 (9.92)	< 0.001
Visceral fat score	5.55 (2.67)	2.13 (1.32)	6.06 (3.26)	11.83 (6.97)	< 0.001
Basal metabolic rate (BMR, kcal)	1416.54 (151.19)	1773.23 (1158.59)	1739.16 (246.26)	1843.06 (416.04)	< 0.001
Waist-to-hip ratio	0.82 (0.08)	0.82 (0.08)	0.84 (0.08)	0.84 (0.23)	0.08
Waist circumference (cm)	82.05 (10.26)	72.27 (6.50)	85.29 (9.97)	92.51 (30.41)	< 0.001
Hip circumference (cm)	100.79 (9.11)	88.62 (8.81)	101.50 (9.26)	112.68 (24.68)	< 0.001
Body temperature (°C)	36.65 (0.47)	36.53 (0.43)	36.43 (0.45)	36.30 (0.45)	0.15
Resting heart rate (bpm)	71.67 (8.95)	72.77 (12.97)	77.93 (12.67)	90.22 (18.89)	0.01
Diastolic blood pressure (mmHg)	84.07 (10.08)	75.38 (9.10)	81.89 (7.22)	93.89 (9.36)	< 0.001
Systolic blood pressure (mmHg)	130.15 (21.21)	113.04 (11.55)	125.89 (13.05)	138.33 (7.71)	< 0.001
Heart Rate (bpm), Lying Position, 5 min	69.33 (6.85)	67.83 (9.74)	68.17 (8.14)	77.18 (7.87)	0.03
HRV (SDNN, ms), lying position, 5 min	31.30 (9.74)	51.78 (19.67)	43.88 (13.29)	20.11 (9.46)	< 0.001
HRV (RMSSD, ms), lying position, 5 min	30.41 (14.01)	51.15 (25.03)	41.66 (16.90)	15.97 (9.53)	< 0.001
HRV (LF, ms^2^), lying position, 5 min	544.88 (352.27)	1527.11 (1134.72)	1148.59 (764.74)	295.95 (224.47)	< 0.001
HRV (HF, ms^2^), lying position, 5 min	435.02 (465.31)	1358.87 (1288.45)	895.79 (780.96)	149.45 (187.31)	< 0.001
HRV (Baevskii index), lying position, 5 min	238.47 (117.39)	117.61 (79.17)	148.28 (85.20)	596.62 (549.83)	< 0.001
Glucose level (mg/dL)	77.37 (10.53)	75.38 (8.47)	82.39 (12.78)	93.33 (43.88)	0.07
CRP (mg/dL)	0.15 (0.10)	0.20 (0.47)	0.36 (0.69)	0.62 (0.54)	< 0.001
AST (U/L)	23.60 (6.54)	20.19 (3.48)	21.57 (6.02)	33.56 (19.25)	0.001
ALT (U/L)	23.86 (9.84)	15.92 (4.67)	24.54 (8.84)	40.89 (22.75)	< 0.001
Gamma-GT (U/L)	19.42 (10.74)	11.77 (4.35)	21.25 (8.79)	61.67 (72.12)	< 0.001
Total cholesterol (mg/dL)	218.84 (44.05)	175.77 (29.08)	196.43 (36.00)	233.89 (47.83)	< 0.001
Triglycerides (mg/dL)	106.10 (35.63)	71.12 (25.77)	116.57 (60.13)	197.56 (62.67)	< 0.001
LDL cholesterol (mg/dL)	134.39 (39.60)	95.35 (23.79)	124.71 (33.77)	155.56 (36.32)	< 0.001
HDL cholesterol (mg/dL)	66.93 (10.71)	69.96 (10.96)	53.46 (11.60)	47.89 (10.45)	< 0.001
Non-HDL cholesterol (mg/dL)	142.14 (34.27)	107.14 (23.73)	140.08 (38.96)	181.63 (41.48)	< 0.001
HbA1c (NGSP, %)	5.34 (0.32)	5.17 (0.29)	5.26 (0.38)	5.59 (1.03)	0.48
Leukocyte Count (10⁹/L)	5.54 (1.26)	5.16 (1.20)	7.05 (1.79)	7.92 (1.95)	< 0.001
Erythrocytes (10^12^/L)	4.58 (0.35)	4.54 (0.33)	4.99 (0.30)	4.74 (0.43)	< 0.001
Hemoglobin (g/dL)	13.79 (1.16)	13.12 (1.11)	14.74 (0.97)	14.61 (1.73)	< 0.001
Hematocrit (%)	40.81 (3.27)	38.93 (2.75)	43.12 (2.45)	42.64 (3.40)	< 0.001
Platelet Count (10⁹/L)	267.89 (69.64)	249.61 (60.43)	275.11 (59.05)	329.56 (84.23)	0.02
Neutrophils (%)	57.18 (8.70)	55.02 (9.79)	58.86 (7.06)	57.22 (8.61)	0.44
Neutrophil Count (10⁹/L)	3.19 (0.92)	2.88 (0.98)	4.18 (1.29)	4.49 (1.24)	< 0.001
Eosinophil Count (10⁹/L)	0.14 (0.08)	0.15 (0.12)	0.18 (0.23)	0.30 (0.22)	0.13
Basophil Count (10⁹/L)	0.05 (0.03)	0.04 (0.02)	0.05 (0.03)	0.07 (0.02)	0.02
Normoblasts (10⁹/L)	0.11 (0.04)	0.10 (0.00)	0.10 (0.00)	0.10 (0.00)	0.85
Lymphocyte Count (10⁹/L)	1.74 (0.56)	1.64 (0.46)	2.06 (0.51)	2.35 (0.83)	0.002
Monocyte Count (10⁹/L)	0.40 (0.10)	0.43 (0.13)	0.54 (0.17)	0.66 (0.25)	< 0.001
Red cell distribution width (RDW, CV, %)	12.83 (0.87)	13.14 (0.87)	12.43 (0.54)	12.96 (0.88)	0.02
Platelet distribution width (PDW, %)	13.17 (2.11)	14.08 (2.30)	12.61 (1.93)	13.00 (1.58)	0.10
Mean platelet volume (MPV, fL)	10.92 (0.96)	11.33 (1.00)	10.68 (0.93)	10.73 (0.86)	0.08
Thyroid-stimulating hormone (TSH, µU/mL)	1.38 (0.71)	1.85 (0.93)	2.39 (1.18)	2.38 (0.91)	< 0.01
Cortisol (µg/dL)	12.11 (3.27)	15.66 (8.32)	13.45 (5.07)	12.94 (4.83)	0.59
IGF-1	119.86 (33.59)	184.92 (52.92)	190.46 (50.14)	119.64 (34.61)	< 0.001
Alpha-1-antitrypsin (g/l)	1.38 (0.16)	1.44 (0.28)	1.38 (0.22)	1.45 (0.15)	0.42
MADRS score	20.70 (8.37)	21.85 ( 6.26)	22.32 (7.06)	28.33 (12.20)	0.10
WHODAS 2.0 score	21.50 (8.78)	23.15 (9.70)	25.59 (8.86)	25.24 (8.43)	0.37
WHO-5 score	11.22 (4.30)	11.97 (4.67)	11.04 (3.62)	10.03 (2.54)	0.77
PHQ-9 score	14.68 (5.49)	13.73 (5.24)	14.98 (4.99)	15.94 (2.63)	0.67
UCLA 3-items score	6.93 (2.30)	7.77 (1.27)	8.64 (2.90)	6.78 (2.05)	0.02*
Lubben social network scale	14.33 (6.39)	12.42 (6.68)	13.43 (5.96)	15.56 (10.34)	0.64
GAD-7 Score	11.25 (5.57)	11.34 (5.15)	11.18 (4.84)	15.00 (4.50)	0.25
PHQ-15 Score	11.76 (5.23)	11.84 (4.88)	10.45 (4.20)	14.33 (7.84)	0.27
Number of siblings	1.30 (0.90)	1.32 (0.93)	1.47 (0.96)	1.56 (0.88)	0.83
Adults in household	1.93 (0.78)	1.76 (0.76)	1.89 (1.07)	2.00 (1.12)	0.91
Education level (1–3)	2.67 (0.62)	2.69 (0.55)	2.64 (0.62)	2.33 (0.71)	0.40
Low education	7.41%	3.9%	7.14%	11.11%	
Medium education	18.52%	23.1%	21.4%	44.4%	
High education	74.1%	73.1%	71.4%	44.4%	
Partner from another ethnicity (%, yes)	14.3%	23.1%	21.9%	7.9%	0.17
Separation in last 12 months (%, yes)	12.2%	15.4%	25.0%	0.0%	0.37
Sport ≥ 1 × per week (%, yes)	59.3%	65.4%	50.00%	22.22%	0.15
Heart attack (%, yes)	3.7%	0.0%	3.6%	11.1%	0.35
Employment in last 12 months (%, yes)	77.3%	84.6%	96.4%	55.6%	0.02*
Biological children (%)	56.6%	15.4%	33.2%	22.22%	0.01*
Migration background (%, yes)	29.6%	30.8%	21.4%	11.1%	0.65
Caring for relatives (%, yes)	3.7%	7.7%	4.1%	0.0%	0.79
Moved during childhood/adolescence (%, yes)	63.5%	76.92%	71.43%	44.4%	0.27
Born in Germany (%, yes)	84.7%	91.8%	88.78%	100.0%	0.55
Mother tongue German (%, yes)	84.7%	91.2%	92.4%	98.4%	0.80
Parents separated (%, yes)	34.4%	57.7%	46.4%	33.3%	0.36
Attempted suicide (%, yes)	25.9%	19.2%	10.7%	33.3%	0.34
Financial difficulties in last 12 months (0–2)	0.22 (0.58)	0.50 (0.81)	0.80 (0.91)	1.25 (0.93)	0.002**
Never	85.1%	69.2%	50.0%	22.2%	
Once	7.4%	11.5%	14.3%	11.1%	
Multiple times	7.4%	19.2%	32.1%	55.6%	

*p < 0.05, **p < 0.01, only significant findings that were not part of the GMM clustering process are marked with a star (*)

*MADRS* Montgomery–Åsberg Depression Rating Scale, measuring the severity of depressive symptoms, *WHODAS 2.0 Score* World Health Organization Disability Assessment Schedule 2.0, *WHO-5 Score* World Health Organization-5 Well-Being Index, *PHQ-9 Score* Patient Health Questionnaire-9, *UCLA 3-items Score* UCLA Loneliness Scale, 3-item version, *PC-PTSD Score* Primary Care PTSD Screen, *GAD-7* Generalized Anxiety Disorder 7-item, *PHQ-15 Score* Patient Health Questionnaire 15, *IGF-1* Insulin-like Growth Factor 1, *CRP* C-Reactive Protein, *TSH* Thyroid Stimulating Hormone, *AST* Aspartate Aminotransferase, *ALT* Alanine Aminotransferase, *LDL* low-densitiy lipoprotein, *HDL* high-densitiy lipoprotein cholesterol, *gamma-GT* gamma-glutamyl transferase

Additionally, higher IGF-1 levels suggested that the *Overweight, Non-Inflammatory* cluster (M: 190.46 SD: 50.14) was significantly metabolically “healthier” compared to the *Immuno-Metabolic* cluster (M: 119.64 SD: 34.61); ANOVA revealed a significant difference between clusters concerning IGF-1 levels (F(3, N = 90) = 15.99, p < 0.001). Post hoc comparisons using the HSD test indicated that the *Overweight, Non-Inflammatory* cluster had significantly higher IGF-1 levels than the *Immuno-Metabolic* cluster (p < 0.001).

While the MADRS Score and most other MADRS items across clusters were not significantly different, there was a significant effect between clusters (H(3) = 10.99, p = 0.01, η^2^ = 0.11) concerning reduced appetite. Post hoc analyses indicated that the *Immuno-Metabolic* cluster had significantly higher scores than participants *in Late Depression* (p = 0.02), the *Adaptive* cluster (p = 0.02), and the *Overweight, Non-Inflammatory* cluster.

The participants in the *Immuno-Metabolic* and *Overweight, Non-Inflammatory* clusters were the least likely to engage in weekly physical activity. Statistics concerning the four clusters are in Table 4.

When comparing the *PC-PTSD Score ratings,* no significant differences among the clusters (H(3) = 4.26, *p* = 0.24, η^2^ = 0.05) were detected.

Financial difficulties within the last year revealed significant differences among clusters (H(3) = 14.37, p < 0.01, η^2^ = 0.14). Post hoc tests showed significant differences between the *Immuno-Metabolic* cluster and Late Depression (*p* < 0.01) and between *Late Depression* and the *Overweight, Non-Inflammatory* cluster (*p* = 0.045), where the *Immuno-Metabolic* cluster, together with the *Overweight, Non-Inflammatory* cluster were most likely to face financial problems.

Patients who were classified among the *Late Depression* cluster were most likely to have parents who had dementia (p = 0.01).

The clusters showed significant differences regarding walking more than 30 min (WHODAS 2.0 Item 7, H(3) = 13.42, p < 0.01, η^2^ = 0.134). Post hoc comparisons showed that the *Adaptive* cluster had the lowest scores (M = 1.31, SD = 0.74), significantly lower than both the *Immuno-Metabolic* cluster (M = 2.49, SD = 1.07, p = 0.01) and the *Overweight, Non-Inflammatory* cluster (M = 2.04, SD = 1.17, p = 0.010), which exhibited the highest and second-highest scores, respectively. Regarding personal hygiene, the *Immuno-Metabolic* cluster was the most impaired (M = 2.90, SD = 1.60), while the *Adaptive* cluster was the least affected (M = 1.38, SD = 0.99, p < 0.001). Post hoc analysis for this domain (WHODAS 2.0 Item 8) using Bonferroni adjustment indicated significant differences, with η^2^ = 0.16, suggesting large effect sizes. Significant group differences were noted for dressing oneself (WHODAS 2.0 Item 9) (H(3) = 9.02, p = 0.05, η^2^ = 0.09). Post hoc analysis revealed significant impairments in the *Late Depression* cluster (M = 1.37, SD = 0.79) compared to the *Immuno-Metabolic* cluster (M = 1.92, SD = 0.90, p = 0.04), as well as in the *Adaptive* cluster (M = 1.42, SD = 0.90, p = 0.05) compared to the *Immuno-Metabolic* cluster (p = 0.045). The *Immuno-Metabolic* cluster and *Overweight, Non-Inflammatory* reported the highest levels of self-care difficulty, highlighting the functional challenges associated with these depressive subtypes.

## Discussion

We address the challenge of improving depression detection in the GP setting, where underdiagnosis potentially contributes to increased morbidity of depression [30].

Hence, we present a novel algorithm based solely on self-rated items from established questionnaires to support GPs in diagnosing depression. The algorithm incorporates a traffic light stratification system: sensitivity and specificity were 93.2% and 93.3% when coupled with a red or green light. For cases flagged with a yellow light, sensitivity and specificity decreased to 77.4% and 78.4%, respectively, while allowing for a diagnostic decision.

The tool is designed as a diagnostic aid for GPs, providing support for further evaluation where necessary. However, GPs must still diagnose depression independently and critically evaluate the model’s precision. There is no data on whether GPs will act upon its predictions.

The PHQ-9, commonly used with a cutoff > 8, demonstrated a BAC of 83.7% in our dataset. Although the *Clinical 15* outperforms the PHQ-9, these results indicate that the PHQ-9 is an adequate alternative when more advanced technical tools are unavailable [31].

A systematic review evaluated studies leveraging electronic health records and ML techniques to predict and diagnose depression. These mentioned algorithms were comparable to established screening tools like the PHQ-9 [32]. To our knowledge, our tool is among the most sensitive and specific tools that have been developed for the GP setting compared to our traffic light risk stratification system [32]. While we plan to make the tool publicly available in the future, it is currently not accessible.

For our final sequential model, *Clinical 15*, we utilized a combination of XGBoost and logistic regression. This approach, while robust, requires computational support and cannot be implemented with pen-and-paper methods. To enable practical application, a digital tool based on an electronic device is essential to ensure the quick calculation of test results.

Within the depressed cohort, we identified an *Immuno-Metabolic* cluster characterized by ANS dysregulation, slight CRP elevation, low IGF-1 levels, metabolic dysregulation, and obesity as determined by BMI as pre-described by Penninx et al. [14, 33], showing that this subtype is also present in GP settings. According to the literature, immuno-metabolic depression (IMD) is often associated with a history of traumatic events. Patients within the *Immuno-Metabolic* cluster reported the highest mean number of depressive episodes, with an average of 10.11 episodes. Further, patients within this cluster had the lowest likelihood of engaging in sports and had more visceral fat, which may contribute to the inflammatory processes. Regular physical activity has been shown to reduce systemic inflammation and improve immune function [34]. Highlighting the potential importance of incorporating tailored lifestyle interventions, such as exercise, alongside medical treatments for these patients. Socioeconomic disadvantage can also increase the risk of immuno-metabolic depression by contributing to stress and unhealthy behaviors [35]; this was evident in our cluster, as patients in this cluster reported financial concerns more frequently.

IGF-1 is involved in neurodevelopment, neurogenesis, and neuronal plasticity [36]. Dysregulation of IGF-1 signaling has been linked to cognitive decline and neuroinflammation, contributing to disease progression in conditions such as depression [37]. Decreased IGF-1 levels may be associated with reduced neuroplasticity. Additionally, depression is linked to chronic stress and hyperactivation of the hypothalamic–pituitary–adrenal (HPA) axis, which can suppress IGF-1 production. Elevated cortisol levels, typical in depression, may contribute to IGF-1 downregulation, exacerbating neuronal vulnerability [38]. Depression is also associated with increased levels of pro-inflammatory cytokines, which can inhibit IGF-1 signaling. Reduced IGF-1 might lead to increased neuroinflammation and neuronal damage [36]. Age and gender influence IGF-1 reference values, and elevated IGF-1 levels, in contrast, are associated with proliferative disorders such as cancer [39]. Additionally, IGF-1 levels increase during pregnancy [40], a life phase that is not typically considered protective against depressive disorders [41]. This suggests that IGF-1 does not have a simple linear effect but interacts in a complex manner with other factors [42]. Further research is needed to elucidate the role of IGF-1 as a biomarker for depression and its potential therapeutic implications [38, 43].

Future improvements to the algorithm could include integrating biological markers as a “third step” to be applied when uncertainty remains after completing all questions of the *Clinical 15* questionnaire. More comprehensive diagnostic capabilities could be developed as similar data sets become available. Additionally, coupling the algorithm with longitudinal data—beyond the currently used cross-sectional data—may pave the way for therapeutic recommendations and prognostic predictions. This progression could provide tailored insights for patients and further support for GPs in managing depression.

Next to the *Immuno-Metabolic* subtype, we found a further subtype of patients with an elevated BMI but lower mean CRP markers than the *Immuno-Metabolic* cluster. This cohort reported significantly higher loneliness, as determined by the UCLA 3-item questionnaire. Interestingly, the IGF-1 values of this cohort were also higher compared to the *Immuno-Metabolic* cluster, showing that the metabolism was not as dysregulated yet. We called this cohort *the Overweight, Non-Inflammatory* cluster.

We named the youngest cluster *Adaptive*, as anthropometric measures and physiological parameters did not reveal deviations from the norm. Compared to the other clusters, we observed a slight elevation in cortisol levels, with a mean value of 15.66 (SD = 8.32); however, this difference was not significant.

We identified a fourth cluster that we named *Late Depression*. This group consisted of older individuals, yet they did not report a higher number of depressive episodes compared to other clusters.

Patients with *IMD* are more likely to have an unsatisfactory response to medication. Celecoxib and other anti-inflammatory treatments are under investigation to improve treatment outcomes [44]. IMD is defined by elevated CRP levels and sometimes clinical symptoms defining atypical depression, although exact thresholds remain an active area of research and are being gradually established [45]. Currently, *IMD* is not an established depressive subtype [13].

For elderly patients with late-onset depression without inflammation or metabolic dysregulation, a watchful waiting approach may be more appropriate than immediate treatment with antidepressants [46]. Regarding Voshaar’s discontinuity hypothesis, he suggests that depressed mood and depressive disorders in later life exist along separate continua, with depressed mood being influenced by age-related factors rather than necessarily progressing into a depressive disorder. Potentially leading to overtreatment if medication is given too quickly [46].

However, severe immuno-metabolic dysregulation in late-life depression is often linked to increased frailty [47], suggesting that different strategies might be necessary.

Currently, only cross-sectional data are available, but longitudinal data will be particularly important and of great interest.

Personalized strategies are essential, as these subtypes are encountered in the GP setting, where referrals to psychiatrists are limited. Further research is crucial to (a) refine diagnostic algorithms and (b) deliver individualized, optimal patient care.

### Limitations

Since we used nested cross-validation to establish our performance metrics, these values should be generalizable. Nevertheless, including external validation data would have been ideal since we can’t exclude biases that might have affected the whole dataset.

Patients were recruited in GP offices using convenience sampling, potentially introducing sampling bias. Additionally, the substantial class imbalance (with one group comprising of 90 participants versus about 491 in the other) was addressed by balancing weights in the XGBoost algorithm; however, such imbalances and the heterogeneity inherent in a naturalistic sample remain potential sources of bias. A critical limitation of our work is the uncertainty regarding how our algorithm’s diagnosis might influence general practitioners’ (GPs) behavior. Consequently, the tool’s true impact cannot be fully estimated without performing a randomized controlled trial.

While the GMM identified four distinct clusters, some clusters were based on relatively small sample sizes, and replication in independent cohorts is necessary to confirm their clinical relevance. Due to its heterogeneity, we did not include medication in the clustering process.

### Conclusion and future work

Our proposed sequential ML model performs better than the PHQ-9 and other published tools. This improved accuracy is likely to result in fewer false positives, making it a more reliable tool for screening depression in GP settings. Additionally, disability, as measured by the WHODAS 2.0, appears to play a critical role in the primary care context, further emphasizing its relevance in depression assessment.

For future work, we are conceptualizing a follow-up study to evaluate our algorithm in a double-blinded, randomized controlled trial. This approach would provide robust evidence regarding its effectiveness and practical utility in clinical environments. Further, we aim to investigate how *Clinical 15* influences GPs’ treatment choices and whether this improves treatment outcomes.

### Supplements

The supplements (S1) provide detailed information about the feature engineering steps. These steps include preprocessing the data to ensure quality and consistency, selecting relevant features to enhance predictive power, and engineering new features based on domain knowledge or exploratory data analysis. Further, the feature importances and corresponding items of all base models can be found in the supplements (S2).

The supplementary sections also provide data concerning the stacked models (S3) and the configuration details of *Clinical 15* (S4). Additionally, we listed the variables used to calculate the GMM (S5), depicted the Q-Q plots before and after transformation (S6), and included the BIC and AIC values used to determine the optimal covariance structure and number of clusters (S7) in the supplementary files.

## Supplementary Information

Below is the link to the electronic supplementary material.

Supplementary file1 (DOCX 134 KB)

## Data Availability

Data is available upon request. The data are securely stored by the POKAL research group.
