# Correction: A multimodal approach to depression diagnosis: insights from machine learning algorithm development in primary care

**DOI:** 10.1007/s00406-025-02058-0

**Published:** 2025-07-02

**Authors:** Julia Eder, Mark Sen Dong, Melanie Wöhler, Maria S. Simon, Catherine Glocker, Lisa Pfeiffer, Richard Gaus, Johannes Wolf, Kadir Mestan, Helmut Krcmar, Nikolaos Koutsouleris, Antonius Schneider, Jochen Gensichen, Richard Musil, Peter Falkai

**Affiliations:** 1 Department of Psychiatry and Psychotherapy, LMU University Hospital, LMU Munich, Nussbaumstraße 7, 80336 Munich, Germany; 2 Graduate Program “POKAL - Predictors and Outcomes in Primary Care” (DFG-GrK 2621), Munich, Germany; 3 German Center for Mental Health (DZPG), Partner Site Munich-Augsburg, Munich, Germany; 4 Chair for Information Systems, Technical University of Munich (TUM), 85748 Garching, Germany; 5 Institute of Psychiatry, Psychology and Neuroscience, King’s College London, London, UK; 6 Institute of General Practice and Health Services Research, School of Medicine, Technical University Munich, Munich, Germany; 7 Institute of General Practice and Family Medicine, Ludwig-Maximilians-University Munich, Munich, Germany; 8 Oberberg Fachklinik Bad Tölz, Bad Tölz, Germany; 9 Max-Planck Institute of Psychiatry, Munich, Germany

**Correction to: European Archives of Psychiatry and Clinical Neuroscience** 10.1007/s00406-025-01990-5

In the article several values published in the text and tables have been corrected to ensure accuracy.

In the “Methods and Design” section, the percentage of participants has been revised from two decimal places to one. The sentence now reads: “Of the remaining 581 participants, 28.4% were recruited at the LMU site, and 71.6% came from various GP offices,” instead of “28.40%” and “71.60%” respectively.

In the “Results” section, The ‘n’ value for outpatient individuals has been corrected to n = 491 from n = 481. The number of participants in the entire cohort who reported having sought professional psychiatric or psychological help has been corrected to n = 271, replacing the previously stated n = 109, which erroneously referred to admissions to psychiatric units or day clinics. The percentage of participants who reported currently taking psychiatric medication has been revised to 16.5% instead of 16.3%.

Additionally, test statistics have been corrected for completeness by including the degrees of freedom. The notation (H = 10.99, p = 0.01, η^2^ = 0.11) now reads (H(3) = 10.99, p = 0.01, η^2^ = 0.11), and (H = 13.42, p < 0.01, η^2^ = 0.134) has been corrected to (H(3) = 13.42, p < 0.01, η^2^ = 0.13).

In the Discussion section, sensitivity and specificity changed to 93.2% and 93.3% instead of 93%.

In addition to the above textual changes, the values and descriptions within the tables have been modified. The corrected Tables 1, 3 and 4 are provided below.

**Table 1:** Descriptive statistics and group comparisons for depressed and non-depressed groups

Variable	Non-depressed (N = 491) M (SD)	Depressed (N = 90) M (SD)	p value
Age (years)	43.47 (14.68)	40.34 (13.10)	0.052
Sex (%, female)	60.1%	68.9%	0.14
Binge drinking (%, yes)	60.2%	50.0%	0.15
Depressive episode before recent episode (%, yes)	10.6%	71.1%	< 0.001***
Currently taking psychiatric medication (%, yes)	8.4%	59.6%	< 0.001***
Number of depressive episodes over lifetime	0.30 (1.39)	5.90 (9.79)	< 0.001***
Total body weight (kg)	73.58 (17.48)	72.67 (17.29)	0.53
Total muscle mass (kg)	52.26 (10.60)	49.57 (9.55)	0.04*
Total body water (kg)	37.17 (8.26)	35.29 (7.42)	0.06
Body mass index (BMI, kg/m^2^)	24.42 (4.91)	24.49 (5.05)	0.76
Body fat percentage (%)	24.39 (7.67)	26.50 (8.57)	0.06
Visceral fat score	5.73 (4.64)	5.29 (4.35)	0.35
Basal metabolic rate (BMR, kcal)	1631.55 (324.97)	1670.96 (706.66)	0.23
Waist-to-hip ratio	0.85 (0.10)	0.83 (0.10)	0.29
Waist circumference (cm)	81.70 (14.60)	81.10 (14.23)	0.97
Hip circumference (cm)	96.47 (11.14)	98.66 (13.63)	0.15
Body temperature (°C)	36.43 (0.44)	36.51 (0.45)	0.07
Resting heart rate (bpm)	70.78 (12.45)	75.79 (13.48)	< 0.001***
Diastolic blood pressure (mmHg)	80.47 (10.41)	81.87 (10.25)	0.13
Systolic blood pressure (mmHg)	124.96 (17.39)	124.70 (17.14)	0.96
Heart rate (bpm), lying position	68.05 (9.81)	69.55 (9.79)	0.13
HRV (SDNN, ms), lying position	45.53 ( 24.56)	39.64 (20.16)	0.10
HRV (RMSSD, ms), lying position	43.17 ( 31.01)	37.76 (24.30)	0.26
HRV (LF, ms^2^), lying position	1351.14 (1452.71)	990.16 (1022.93)	0.02*
HRV (HF, ms^2^), lying position	972.55 (1380.75)	799.09 (1077.74)	0.36
HRV (Baevskii index), lying position	170.95 (247.96)	224.24 (271.75)	0.03*
Glucose level (mg/dL)	75.41 (14.06)	80.06 (17.60)	0.001**
CRP (mg/dL)	0.23 (0.70)	0.28 (0.51)	0.29
AST (U/L)	23.97 (7.76)	22.99 (8.73)	0.10
ALT (U/L)	23.36 (13.08)	23.50 (12.48)	0.89
Gamma-GT (U/L)	20.86 (19.87)	22.14 (27.16)	0.88
Total cholesterol (mg/dL)	192.32 (40.60)	201.01 (42.93)	0.09
Triglycerides (mg/dL)	93.71 (52.77)	108.19 ( 57.55)	0.02*
LDL-cholesterol (mg/dL)	114.64 (34.68)	122.36 ( 38.52)	0.10
HDL-cholesterol (mg/dL)	61.76 (15.87)	61.78 (13.81)	0.73
Non-HDL cholesterol (mg/L)	132.53 (39.82)	135.22 (44.39)	0.85
HbA1c (NGSP, %)	5.29 (0.53)	5.29 (0.46)	0.53
Leukocyte count (10⁹/L)	5.49 (1.50)	6.15 (1.79)	0.002**
Erythrocyte count (10^12^/L)	4.72 ( 0.44)	4.71 ( 0.39)	0.83
Hemoglobin (g/dL)	14.01 (1.59)	13.98 (1.33)	0.66
Hematocrit (%)	41.35 (3.48)	41.19 (3.35)	0.70
Platelet count (10^9^/L)	251.03 (62.11)	271.39 (68.66)	0.02*
Neutrophil count (10^9^/L)	3.09 (1.14)	3.55 (1.26)	< 0.001***
Monocyte count (10^9^/L)	0.43 (0.13)	0.48 (0.17)	0.03*
Eosinophil count (10^9^/L)	0.16 (0.15)	0.17 (0.17)	0.92
Basophil count (10^9^/L)	0.04 (0.02)	0.05 (0.02)	0.02*
Normoblast count (10^9^/L)	0.10 (0.00)	0.10 (0.02)	0.35
Lymphocyte count (10^9^/L)	1.75 (0.54)	1.88 (0.59)	0.07
Monocyte count (10^9^/L)	0.43 (0.13)	0.48 (0.17)	0.03
TSH (µU/mL)	2.03 (1.43)	1.93 (1.04)	0.90
Cortisol (µg/dL)	13.88 (7.03)	14.07 (6.67)	0.71
IGF-1 (ng/mL)	163.31 (59.65)	160.87 (56.05)	0.83
Alpha-1-antitrypsin (g/L)	1.36 (0.22)	1.41 ( 0.22)	0.049
MADRS score	4.72 (4.93)	22.30 (8.03)	< 0.001***
WHODAS 2.0 score	7.46 (8.09)	24.01 (10.05)	< 0.001***
WHO-5 score	18.15 (4.84)	11.23 (4.11)	< 0.001***
PHQ-9 score	4.77 (3.51)	14.68 (5.18)	< 0.001***
UCLA 3-items score	5.27 (1.78)	7.69 *(*2.34)	< 0.001***
PC-PTSD score	0.57 (1.10)	1.98 (1.84)	< 0.001***
Lubben social network scale	14.35 (4.75)	13.58 (6.80)	< 0.02*
GAD-7 score	4.20 (3.74)	11.59 (5.21)	< 0.001***
PHQ-15 score	5.82 (3.84)	11.64 (5.34)	< 0.001***
Number of siblings	1.30 ( 0.86)	1.39 (0.94)	0.40
Adults in household	1.93 (0.74)	1.88 (0.93)	0.17
Partner from another ethnicity (%, yes)	21.9%	18.5%	0.70
Separation in the last 12 months (%, yes)	6.7%	15.9%	0.01*
Sport ≥ 1 × per week (%, yes)	77.2%	54.4%	< 0.001***
Heart attack (%, yes)	5.9%	3.4%	0.49
Employment in the last 12 months (%, yes)	86.2%	82.8%	0.49
Biological children (%, yes)	44.1%	34.1%	0.10
Migration background (%, yes)	23.4%	25.8%	0.72
Caring for relatives (%, yes)	9.3%	4.6%	0.21
Moved during childhood/adolescence (%, yes)	60.0%	68.2%	0.19
Born in Germany (%, yes)	87.0%	89.3%	0.69
Mother tongue German (%, yes)	90.1%	90.5%	0.99
Parents separated (%, yes)	26.1%	44.9%	< 0.001***
Attempted suicide (%, yes)	2.5%	20.2%	< 0.001***

*p < 0.05, **p < 0.01, ***p < 0.001 MADRS: Montgomery–Åsberg Depression Rating Scale, measuring the severity of depressive symptoms

*WHODAS 2.0 Score* World Health Organization Disability Assessment Schedule 2.0 12-item score, *WHO-5 Score* World Health Organization-5 Well-Being Index, *PHQ-9 Score* Patient Health Questionnaire-9, *UCLA 3-items Score* UCLA Loneliness Scale, 3-item version, *PC-PTSD Score* Primary Care PTSD Screen, *GAD-7* Generalized Anxiety Disorder 7-item, *PHQ-15 Score*: Patient Health Questionnaire 15, *IGF-1* Insulin-like Growth Factor 1, *CRP* C-reactive protein, *TSH* thyroid stimulating hormone, *AST* aspartate aminotransferase, *ALT* alanine aminotransferase, *LDL* low-density lipoprotein, *HDL* high-density lipoprotein cholesterol, *gamma-GT* gamma-glutamyl transferase

**Table 3:** The performance of sequential model depicting the traffic light system and the probabilities of correct and incorrect disease classification

Model name	Total		High certainty groups		Low certainty groups	
			Red traffic light	Green traffic light	Yellow traffic light	
	Depression	HC	Depression	HC	Depression	HC
Clinical 5 (first step)	76/90 (84.4%)	424/491 (86.4%)	38/45 (84.4%)	219/235 (93.2%)	38/45 (84.4%)	205/256 (80.1%)
Clinical 15 (second step)	**79/90 (87.8%)**	**436/491 (88.8%)**	**55/59 (93.2%)**	**320/343 (93.3%)**	24/31 (77.4%)	116/148 (78.4%)

The table shows the number of correctly classified instances versus the total number of true instances for both classes in the clinical sequential system

*HC* healthy control

**Table 4:** Shows descriptive statistics as well as group comparisons of clusters that were found through GMM

	Late depression, n = 27, M (SD)	Adaptive, n = 26, M (SD)	Overweight non-inflammatory, n = 28, M (SD)	Immuno-metabolic, n = 9, M (SD)	p value
Age at study Inclusion (years)	50.76 (10.85)	33.20 (9.97)	35.39 (11.05)	45.27 (11.77)	< 0.001
Sex (%, female)	88.9	80.8	42.9	55.6	0.001
Binge drinking (%, yes)	18.5	49.5	65.8	52.4	0.001**
Depressive episode before recent episode (%, yes)	70.4	69.2	82.1	44.4	0.20
Currently Taking Psychiatric Medication (%, yes)	70.4	40.7	64.3	66.7	0.12
Number of depressive episodes over lifetime	4.23 (4.11)	4.23 (3.47)	5.30 (8.87)	9.63 (15.32)	0.35
Total body weight (kg)	67.76 (9.45)	62.41 (8.01)	80.33 (12.89)	94.91 (28.89)	< 0.001
Total muscle mass (kg)	44.31 (5.22)	46.05 (5.95)	55.86 (8.42)	57.44 (11.93)	< 0.001
Total body water (kg)	31.48 (3.38)	31.89 (3.94)	40.19 (6.15)	42.42 (10.52)	< 0.001
Body mass index (BMI, kg/m^2^)	24.01 (3.30)	21.57 (2.37)	25.38 (3.92)	31.51 (8.45)	< 0.001
Body fat percentage (%)	28.70 (7.58)	22.07 (5.14)	26.14 (8.60)	33.84 (9.92)	< 0.001
Visceral fat score	5.55 (2.67)	2.13 (1.32)	6.06 (3.26)	11.83 (6.97)	< 0.001
Basal metabolic rate (BMR, kcal)	1416.54 (151.19)	1773.23 (1158.59)	1739.16 (246.26)	1843.06 (416.04)	< 0.001
Waist-to-hip ratio	0.82 (0.08)	0.82 (0.08)	0.84 (0.08)	0.84 (0.23)	0.08
Waist circumference (cm)	82.05 (10.26)	72.27 (6.50)	85.29 (9.97)	92.51 (30.41)	< 0.001
Hip circumference (cm)	100.79 (9.11)	88.62 (8.81)	101.50 (9.26)	112.68 (24.68)	< 0.001
Body temperature (°C)	36.65 (0.47)	36.53 (0.43)	36.43 (0.45)	36.30 (0.45)	0.15
Resting heart rate (bpm)	71.67 (8.95)	72.77 (12.97)	77.93 (12.67)	90.22 (18.89)	0.01
Diastolic blood pressure (mmHg)	84.07 (10.08)	75.38 (9.10)	81.89 (7.22)	93.89 (9.36)	< 0.001
Systolic blood pressure (mmHg)	130.15 (21.21)	113.04 (11.55)	125.89 (13.05)	138.33 (7.71)	< 0.001
Heart rate (bpm), lying position	69.33 (6.85)	67.83 (9.74)	68.17 (8.14)	77.18 (7.87)	0.03
HRV (SDNN, ms), lying position	31.30 (9.74)	51.78 (19.67)	43.88 (13.29)	20.11 (9.46)	< 0.001
HRV (RMSSD, ms), lying position	30.41 (14.01)	51.15 (25.03)	41.66 (16.90)	15.97 (9.53)	< 0.001
HRV (LF, ms^2^), Lying position	544.88 (352.27)	1527.11 (1134.72)	1148.59 (764.74)	295.95 (224.47)	< 0.001
HRV (HF, ms^2^), Lying position	435.02 (465.31)	1358.87 (1288.45)	895.79 (780.96)	149.45 (187.31)	< 0.001
HRV (Baevskii Index), lying position	238.47 (117.39)	117.61 (79.17)	148.28 (85.20)	596.62 (549.83)	< 0.001
Glucose level (mg/dL)	77.37 (10.53)	75.38 (8.47)	82.39 (12.78)	93.33 (43.88)	0.07
CRP (mg/dL)	0.15 (0.10)	0.20 (0.47)	0.36 (0.69)	0.62 (0.54)	< 0.001
AST (U/L)	23.60 (6.54)	20.19 (3.48)	21.57 (6.02)	33.56 (19.25)	0.001
ALT (U/L)	23.86 (9.84)	15.92 (4.67)	24.54 (8.84)	40.89 (22.75)	< 0.001
Gamma-GT (U/L)	19.42 (10.74)	11.77 (4.35)	21.25 (8.79)	61.67 (72.12)	< 0.001
Total cholesterol (mg/dL)	218.84 (44.05)	175.77 (29.08)	196.43 (36.00)	233.89 (47.83)	< 0.001
Triglycerides (mg/dL)	106.10 (35.63)	71.12 (25.77)	116.57 (60.13)	197.56 (62.67)	< 0.001
LDL cholesterol (mg/dL)	134.39 (39.60)	95.35 (23.79)	124.71 (33.77)	155.56 (36.32)	< 0.001
HDL cholesterol (mg/dL)	66.93 (10.71)	69.96 (10.96)	53.46 (11.60)	47.89 (10.45)	< 0.001
Non-HDL cholesterol (mg/dL)	142.14 (34.27)	107.14 (23.73)	140.08 (38.96)	181.63 (41.48)	< 0.001
HbA1c (NGSP, %)	5.34 (0.32)	5.17 (0.29)	5.26 (0.38)	5.59 (1.03)	0.48
Leukocyte count (10^9^/L)	5.54 (1.26)	5.16 (1.20)	7.05 (1.79)	7.92 (1.95)	< 0.001
Erythrocyte Count (10^12^/L)	4.58 (0.35)	4.54 (0.33)	4.99 (0.30)	4.74 (0.43)	< 0.001
Hemoglobin (g/dL)	13.79 (1.16)	13.12 (1.11)	14.74 (0.97)	14.61 (1.73)	< 0.001
Hematocrit (%)	40.81 (3.27)	38.93 (2.75)	43.12 (2.45)	42.64 (3.40)	< 0.001
Platelet count (10^9^/L)	267.89 (69.64)	249.61 (60.43)	275.11 (59.05)	329.56 (84.23)	0.02
Neutrophils (%)	57.18 (8.70)	55.02 (9.79)	58.86 (7.06)	57.22 (8.61)	0.44
Neutrophil count (10^9^/L)	3.19 (0.92)	2.88 (0.98)	4.18 (1.29)	4.49 (1.24)	< 0.001
Eosinophil count (10^9^/L)	0.14 (0.08)	0.15 (0.12)	0.18 (0.23)	0.30 (0.22)	0.13
Basophil count (10^9^/L)	0.05 (0.03)	0.04 (0.02)	0.05 (0.03)	0.07 (0.02)	0.02
Normoblast count (10^9^/L)	0.11 (0.04)	0.10 (0.00)	0.10 (0.00)	0.10 (0.00)	0.85
Lymphocyte count (10^9^/L)	1.74 (0.56)	1.64 (0.46)	2.06 (0.51)	2.35 (0.83)	0.002
Monocyte count (10^9^/L)	0.40 (0.10)	0.43 (0.13)	0.54 (0.17)	0.66 (0.25)	< 0.001
Red cell distribution width (RDW, CV, %)	12.83 (0.87)	13.1 (0.87)	12.43 (0.54)	12.96 (0.88)	0.02
Platelet distribution width (PDW, %)	13.17 (2.11)	14.08 (2.30)	12.61 (1.93)	13.00 (1.58)	0.10
Mean platelet volume (MPV, fL)	10.92 (0.96)	11.33 (1.00)	10.68 (0.93)	10.73 (0.86)	0.08
Thyroid-stimulating hormone (TSH, µU/mL)	1.38 (0.71)	1.85 (0.93)	2.39 (1.18)	2.38 (0.91)	< 0.01
Cortisol (µg/dL)	12.11 (3.27)	15.66 (8.32)	13.45 (5.07)	12.94 (4.83)	0.58
IGF-1	119.86 (33.59)	184.92 (52.92)	190.46 (50.14)	119.64 (34.61)	< 0.001
Alpha-1-antitrypsin (g/l)	1.38 (0.16)	1.44 (0.28)	1.38 (0.22)	1.45 (0.15)	0.42
MADRS score	20.70 (8.37)	21.85 ( 6.26)	22.32 (7.06)	28.33 (12.20)	0.10
WHODAS 2.0 score	21.50 (8.78)	23.15 (9.70)	25.59 (8.86)	25.24 (8.43)	0.37
WHO-5 score	11.22 (4.30)	11.97 (4.67)	11.04 (3.62)	10.03 (2.54)	0.77
PHQ-9 score	14.68 (5.49)	13.73 (5.24)	14.98 (4.99)	15.94 (2.63)	0.67
UCLA 3-items score	6.93 (2.30)	7.77 (1.27)	8.64 (2.90)	6.78 (2.05)	0.02*
Lubben social network scale	14.33 (6.39)	12.42 (6.68)	13.43 (5.96)	15.56 (10.34)	0.64
GAD-7 score	11.25 (5.57)	11.34 (5.15)	11.18 (4.84)	15.00 (4.50)	0.25
PHQ-15 score	11.76 (5.23)	11.84 (4.88)	10.45 (4.20)	14.33 (7.84)	0.27
Number of siblings	1.30 (0.90)	1.32 (0.93)	1.47 (0.96)	1.56 (0.88)	0.83
Adults in household	1.93 (0.78)	1.76 (0.76)	1.89 (1.07)	2.00 (1.12)	0.91
Education level (1–3)	2.67 (0.62)	2.69 (0.55)	2.64 (0.62)	2.33 (0.71)	0.40
Low education	7.4%	3.9%	7.1%	11.1%	
Medium education	18.5%	23.1%	21.4%	44.4%	
High education	74.1%	73.1%	71.4%	44.4%	
Partner from another ethnicity (%, yes)	14.3%	23.1%	21.9%	7.9%	0.17
Separation in last 12 months (%, yes)	12.2%	15.4%	25.0%	0.0%	0.37
Sport ≥ 1 × per week (%, yes)	59.3%	65.4%	50.0%	22.2%	0.15
Heart attack (%, yes)	3.7%	0.0%	3.6%	11.1%	0.35
Employment in last 12 months (%, yes)	77.3%	84.6%	96.4%	55.6%	0.02*
Biological children (%)	56.6%	15.4%	33.2%	22.2%	0.01*
Migration background (%, yes)	29.6%	30.8%	21.4%	11.1%	0.65
Caring for relatives (%, yes)	3.7%	7.7%	4.1%	0.0%	0.79
Moved during childhood/adolescence (%, yes)	63.5%	76.9%	71.4%	44.4%	0.27
Born in Germany (%, yes)	84.7%	91.8%	88.8%	100.0%	0.55
Mother tongue German (%, yes)	84.7%	91.2%	92.4%	98.4%	0.80
Parents separated (%, yes)	34.4%	57.7%	46.4%	33.3%	0.36
Attempted suicide (%, yes)	25.9%	19.2%	10.7%	33.3%	0.34
Financial difficulties in last 12 months (0–2)	0.22 (0.58)	0.50 (0.81)	0.80 (0.91)	1.25 (0.93)	0.002**
Never	85.1%	69.2%	50.0%	22.2%	
Once	7.4%	11.5%	14.3%	11.1%	
Multiple times	7.4%	19.2%	32.1%	55.6%	

*p < 0.05, **p < 0.01, only significant findings that were not part of the GMM clustering process are marked with a star (*)

*MADRS* Montgomery–Åsberg Depression Rating Scale, measuring the severity of depressive symptoms, *WHODAS 2.0 Score* World Health Organization Disability Assessment Schedule 2.0, *WHO-5 Score* World Health Organization-5 Well-Being Index, *PHQ-9 Score* Patient Health Questionnaire-9, *UCLA 3-items Score* UCLA Loneliness Scale, 3-item version, *PC-PTSD Score*: Primary Care PTSD Screen, *GAD-7* Generalized Anxiety Disorder 7-item, *PHQ-15 Score* Patient Health Questionnaire 15, *IGF-1* Insulin-like Growth Factor 1, *CRP* C-reactive protein, *TSH* thyroid stimulating hormone, *AST* aspartate aminotransferase, *ALT* alanine aminotransferase, *LDL* low-density lipoprotein, *HDL* high-density lipoprotein cholesterol, *gamma-GT* gamma-glutamyl transferase

The original article has been corrected.

