# Supplementary material for: A multimodal approach to depression diagnosis: insights from machine learning algorithm development in primary care
Source: Eur Arch Psychiatry Clin Neurosci. 2025 Mar 10;276(2):407–20. doi: 10.1007/s00406-025-01990-5 (PMC12953261; doi:10.1007/s00406-025-01990-5)
Supplement: Supplementary file 1 — Supplementary file1 (DOCX 134 KB) [file 406_2025_1990_MOESM1_ESM.docx]

# Supplements

## (S1) Feature engineering steps:

### Biographic Data

In the feature engineering process, several transformations were applied to the dataset to prepare it for the machine learning pipeline, which we will describe in detail in this document. Initially, various items were renamed for clarity and easier interpretation.

- 'PSY_BBM_SDI_007_CB_01' was renamed to 'derzeitiger Familienstand'.
- 'PSY_BBM_SDI_009_DD_01' became 'Anzahl Erwachsene im Haushalt'.
- 'PSY_BBM_SDI_015_RB_01' was changed to 'Höchster Schulabschluss'.
- 'PSY_BBM_SDI_020_FT_01' was renamed to 'Arbeitsstunden pro Woche'.
- 'PSY_BBM_SDI_024_DD_01_old' became 'Muttersprache'.
- 'PSY_BBM_SDI_025_DD_01' was changed to 'Größe aktueller Wohnort'.
- 'PSY_BBM_SDI_019_DD_02' was updated to '1. Arbeitsmarkt'.
- 'PSY_BBM_SDI_027_DD_02' was renamed to 'im Alter von 0-5 Jahren umgezogen'.
- 'PSY_BBM_SDI_027_DD_03' became 'im Alter von 5-10 Jahren umgezogen'.
- 'PSY_BBM_SDI_027_DD_04' was changed to 'im Alter von 10-15 Jahren umgezogen'.
- 'PSY_BBM_DRG_006_DD_02' was renamed to 'Cannabis / THC-Produkte'.
- 'PSY_BBM_PVG_007_DD_02' became 'Haben sich Ihre Eltern getrennt?'.
- 'PSY_BBM_PVG_009_DD_02' was updated to 'Wie viele Geschwister haben Sie?'.
- 'PSY_BBM_PVG_009_DD_03' was changed to 'Wie viele Halbgeschwister haben Sie?'.
- 'PSY_BBM_PVG_RB_02' was renamed to 'Angststörung'.
- 'PSY_BBM_PVG_RB_03' became 'Depression'.
- 'PSY_BBM_PVG_010_RA_04' was updated to 'Zwangserkrankung'.
- 'PSY_BBM_PVG_010_RB_05' was renamed to 'Alkohol- oder Drogenabhängigkeit'.
- 'PSY_BBM_PVG_010_RB_06' became 'Medikamentenabhängigkeit'.
- 'PSY_BBM_PVG_010_RB_07' was updated to 'Schizophrenie oder Psychose'.
- 'PSY_BBM_PVG_010_RB_08' was renamed to 'Manisch-depressive (bipolare) Erkrankung'.
- 'PSY_BBM_PVG_010_RB_09' became 'Demenz / demenzielle Erkrankung'.
- 'PSY_BBM_PVG_010_RB_10' was changed to 'Autismus'.
- 'PSY_BBM_PVG_010_RB_11' was renamed to 'Geistige Behinderung'.
- 'PSY_BBM_PVG_010_RB_12' became 'Nicht definierte psychische Erkrankung / starker Verdacht auf psychische Erkrankung'.
- 'PSY_BBM_PVG_010_RB_13' was renamed to 'Suizide in ihrer Verwandtschaft'.
- 'PSY_BBM_PVG_012_RB_01' became 'Hatte ein Verwandter von Ihnen vor seinem 60. Lebensjahr einen Herzinfarkt?'.
- 'PSY_BBM_PVG_017_DD_01' was changed to 'Haben sie schon mal versucht sich das Leben zu nehmen?'.
- 'PSY_BBM_PVG_010_CB_02' was renamed to 'Angststörung ist aufgetreten bei:'.
- 'PSY_BBM_PVG_010_CB_03' became 'Depression ist aufgetreten bei:'.
- 'PSY_BBM_PVG_010_CB_04' was updated to 'Zwangserkrankung ist aufgetreten bei:'.
- 'PSY_BBM_PVG_010_CB_05' was renamed to 'Alkohol- oder Drogenabhängigkeit ist aufgetreten bei:'.
- 'PSY_BBM_PVG_010_CB_06' became 'Medikamentenabhängigkeit ist aufgetreten bei:'.
- 'PSY_BBM_PVG_010_CB_07' was updated to 'Schizophrenie oder Psychose ist aufgetreten bei:'.
- 'PSY_BBM_PVG_010_CB_08' was renamed to 'Manisch-depressive (bipolare) Erkrankung ist aufgetreten bei:'.
- 'PSY_BBM_PVG_010_CB_09' became 'Demenz / demenzielle Erkrankung ist aufgetreten bei:'.
- 'PSY_BBM_PVG_010_CB_10' was changed to 'Autismus ist aufgetreten bei:'.
- 'PSY_BBM_PVG_010_CB_11' was renamed to 'Geistige Behinderung ist aufgetreten bei:'.
- 'PSY_BBM_PVG_010_CB_12' became 'Nicht definierte psychische Erkrankung / starker Verdacht auf psychische Erkrankung ist aufgetreten bei:'.
- 'PSY_BBM_PVG_010_CB_13' was updated to 'Suizide in der Verwandtschaft ist aufgetreten bei:'.
- 'PSY_BBM_PVG_012_CB_01' was renamed to 'Herzinfarkt unter 60 Jahren ist aufgetreten bei:'.
- 'PSY_BBM_SDI_019_DD_01' was changed to '
- 'PSY_BBM_SDI_022_DD_01' was renamed to 'Migrationshintergrund'
- 'PSY_BBM_SDI_014_DD_01' is now named: 'Versorgen_Sie_pflegebeduerftige_Angehoerige'
- 'PSY_BBM_SDI_027_DD_01'] was updated to 'In_Ihrer_Kindheit/Jugend_umgezogen'
- ﻿‚PSY_BBM_DRG_005_DD_03' was changed to ﻿'Binge_Drinking_1_nie_5_fast_taegl'
- ‘PSY_BBM_DRG_006_DD_01' is now named 'andere_Substanzen'
- 'PSY_BBM_TDF_004_DD_01' was renamed to 'Lubben_1',
- 'PSY_BBM_TDF_004_DD_02' was changed to 'Lubben_2',
- 'PSY_BBM_TDF_004_DD_03' became 'Lubben_3',
- 'PSY_BBM_TDF_005_DD_01' was updated to'Lubben_4',
- 'PSY_BBM_TDF_005_DD_02' now reads 'Lubben_5',
- 'PSY_BBM_TDF_005_DD_03' was renamed to 'Lubben_6',

Items that contained categorical strings were transformed into numeric values. For example: "ja" was converted to 1, "nein" to 0, and any empty strings were replaced with NaN values to represent missing data.

For the variables "Born_in_Germany" (initially named: ﻿'PSY_BBM_SDI_023_DD_01_old') and "Muttersprache_DEU," (initially named: ﻿“PSY_BBM_SDI_024_DD_01_old') responses such as 'D' and 'DEU' were converted to 1, while all other values were assigned a 0. Similar to the other transformations, empty strings in these variables were also replaced with NaN.

The "derzeitiger Familienstand" variable was transformed into a binary format to facilitate its use in a machine learning model. Specifically, statuses indicating a married or partnered situation ("HEIRAT" or "PAAR") were recoded as 1, while statuses indicating being single, widowed, divorced, or separated ("LEDIG," "WIT," "SCHEIDUNG," or "Getrennt") were recoded as 0. The function also handled cases where the marital status was missing, returning NaN to preserve the representation of missing data. If someone wrote “HEIRAT,getrennt” it was handled as 0. These participants were legally still married, but already living separated from their partner. A new column, ‘derzeitiger_Familienstand_rec’, which contains the recoded binary values.

The variable "Alter bei Studieneinschluss" was derived by calculating the difference between the birthdate of the individual and the date of study inclusion, providing an accurate measure of age at the time of entering the study.

Additionally, for the items "Wie viele Geschwister haben Sie?" and "Wie viele Halbgeschwister haben Sie?", the reported numbers were adjusted to accurately reflect the total number of siblings.

﻿The item ‘Anzahl Erwachsene im Haushalt' was clipped above 4 and treated as 4 since higher values were too sparse.

Further, we recoded the item 'Cannabis / THC-Produkte' which reflect the requency of self-reported THC product usage. If the reported cannabis use was 2 or more, the we assigned a value of 2. If cannabis use was reported as 1, it assigns a value of 1. If no other substance use was reported (indicated by the value 'nein' for the variable andere_Substanzen), it assigns a value of 0. If neither condition is met, it returns NaN to indicate missing or unclear data. This function was applied to create a new column, ‘Cannabis_n’, which stores the recoded values.

Next, smoking habits were transformed into specific numeric values. If the smoking habit value is 5 or greater (daily smoker), it assigns a value of 7. For values of 4, it assigns 1(smoked for the last time a week ago); for values between 2 and 3, it assigns 0.02083 (ex- smoker either more than a month or a year ago). If the smoking habit is reported as 1 (never smoked), it assigns a value of 0. For any other condition, it returns NaN. This transformation was applied to create a new column, ‘Zigarettenkonsum’, which stores the recoded smoking habits.

Additionally, the variable ‘Binge_Drinking’ was created (original variable: Binge_Drinking_1_nie_5_fast_taegl). If the value was 1 (indicating never engaging in binge drinking), the function assigns a value of 0. For any other valid entry, it assigns a value of 1. If the data is missing or was unclear, ‘nan’ was assigned

Additionally, an ordinal encoding was applied to the educational level variable (which was initially categorical), 'Höchster Schulabschluss,' to convert it into a more meaningful numeric format. This was done by defining an ordinal mapping where 'Low Education' was mapped to 1 (consisting out of the items: ), 'Medium Education' to 2 (consisting out of the items: ), 'High Education' to 3 (consisting out of the items: ), and 'Unknown' was treated as NaN. This encoding was applied to the processed data under the new column 'Education_Level'. The dataset was then refined, focusing on these encoded and engineered features for further analysis in the machine learning pipeline.

The variable "Arbeitsstunden pro Woche" was alre categorized into discrete bins based on custom_cut_offs. These bins were defined to group the data into ranges: 0-10, 10-20, 20-35, 35-45, and 45-120 hours per week. The result of this binning process was stored in a new column called Arbeitsstunden_Bins.

Following this, a bin_mapping dictionary was created to assign ordinal values to each of the defined bins. For instance, the interval 0-10 hours was mapped to 1, 10-20 hours to 2, and so forth, up to 45-120 hours, which was mapped to 5. This mapping was applied to the ‘Arbeitsstunden_Bins’ column, resulting in the creation of a new column called ‘Arbeitsstunden_Ordinal’, where each bin was replaced with its corresponding ordinal value. This transformation makes the data easier to analyze in ordinal terms.

Concerning the Items containing information about the family history like: “Angststörung ist aufgetreten bei:” - we generated new columns indicating which family member was affected. For example, if a disorder was indicated by the code '1,' a new column was created for the father (e.g., 'Angststörung_Vater'), whereas a code '0' would result in a column for the mother (e.g., 'Angststörung_Mutter'). Similarly, if the disorder affected the respondent's own child (code '3'), a column like 'Angststörung_eig_Kind' was created. Codes '3,' '4,' '5,' or '6' led to the creation of a column for siblings (e.g., 'Angststörung_Geschwister'), and codes '7' or '8' resulted in a column for other relatives (e.g., 'Angststörung_andere'). To address sparsity in the data, some columns were merged. For instance, the columns representing the diagnosis of an anxiety disorder or an OCD in one or both parents were combined into a new column, 'Angst+Zwang_Eltern,' where the values were clipped between 0 and 1. Similarly, addiction-related columns were combined into 'addiction_parents,' and severe mental disorders affecting parents were merged into 'Severe_mental_disorder_parents,' For the latter item the parents were reported to have suffered from bipolar disorder, schizophrenia or conducted suicide. The variable 'Suicides_close_relatives' was created by merging multiple columns representing suicides in various family members.

The PHQ-15 items named 'PSY_Pokal_PHQ-15_1', 'PSY_Pokal_PHQ-15_2' … 'PSY_Pokal_PHQ-15_15' contained Strings that that either endet with 1,2 or 0 – inidicating the value for each item. Therefore the items were transformed to an integer value accordingly (﻿'PSY_Pokal_PHQ-15' 🡪 0, 'PSY_Pokal_PHQ-15_1'🡪1, 'PSY_Pokal_PHQ-15_2'🡪 2)

The remapping of the Lubben scale was carried out to align with the scoring methodology outlined in the original paper. The process involved reversing the scale of responses in six specific columns: 'Lubben_1', 'Lubben_2', 'Lubben_3', 'Lubben_4', 'Lubben_5', and 'Lubben_6'. A mapping dictionary was defined where the original values were reversed: 0 was mapped to 5, 1 to 4, 2 to 3, 3 to 2, 4 to 1, and 5 to 0. This remapping was applied to each of the six Lubben columns.

After the remapping, a sum score ‘Lubben_gesamt’, was created. This total score (Lubben_gesamt) represents the overall Lubben Social Network Scale score, reflecting the participant's level of social engagement.

### Selfrating Data

The Selfrating_data consisted out of the following questionnaire set that were given to the patients after they agreed on participating in the study: PHQ9, UCLA 3- items questionnaire, WHODAS, the PC-PTSD and the WHO5.

The values in the WHODAS columns, which initially ranged from 0 to 4, were incremented by 1 to align them with the original coding of the WHODAS questionnaire.

ADD WHODAS clipped !

After this adjustment, a total WHODAS score (WHODAS_Total_Score) was calculated by summing the values across all WHODAS items. To further process the data, each WHODAS item was then clipped to a maximum value of 4, ensuring that no response exceeded this upper limit. This process did not interfere with the calculation of the sumscores.

Next, an extended mapping was applied to rename the columns from their original codes to more intuitive names corresponding to the specific items of the PHQ9, UCLA, WHO5, and PC-PTSD questionnaires.

'PSY_BBM_TDF_003_DD_02' 🡪 'UCLA1' ,

'PSY_BBM_TDF_003_DD_03'🡪 'UCLA2' ,

'PSY_BBM_TDF_003_DD_04'🡪 'UCLA3' ,

'PSY_BBM_TDF_006_DD_02'🡪 'WHO5_1',

'PSY_BBM_TDF_006_DD_03'🡪'WHO5_2',

'PSY_BBM_TDF_006_DD_04'🡪 'WHO5_3',

'PSY_BBM_TDF_006_DD_05'🡪 'WHO5_4',

'PSY_BBM_TDF_006_DD_06'🡪 'WHO5_5',

'PSY_BBM_TDF_007_DD_02'🡪 'PHQ9_1',

'PSY_BBM_TDF_007_DD_03'🡪 'PHQ9_2',

'PSY_BBM_TDF_007_DD_04'🡪 'PHQ9_3',

'PSY_BBM_TDF_007_DD_05'🡪 'PHQ9_4',

'PSY_BBM_TDF_007_DD_06'🡪'PHQ9_5',

'PSY_BBM_TDF_007_DD_07'🡪 'PHQ9_6',

'PSY_BBM_TDF_007_DD_08'🡪 'PHQ9_7',

'PSY_BBM_TDF_007_DD_09'🡪 'PHQ9_8',

'PSY_BBM_TDF_007_DD_10'🡪 'PHQ9_9'

This renaming facilitated easier interpretation and analysis of the data.

After renaming, sum scores for the questionnaires were generated. For the WHO5 questionnaire, 1 was subtracted from each item’s original value to comply with the original paper of this questionnaire, and the sum score (WHO5_sum_score) was calculated by summing the adjusted values across all WHO5 items. Similar sum scores were calculated for the UCLA 3-items questionnaire (UCLA_3-items_score), PHQ9 (PHQ9_score), and the PC-PTSD (PC-PTSD_score) by summing the respective item scores within each questionnaire.

### Heart Rate Variability Data

For the Heart Rate Variability (HRV) Model the following items were used : ﻿'PSY_Pokal_HRV_Gesamtdurchschnitt','PSY_Pokal_HRV_SDNN_gesamt',

'PSY_Pokal_HRV_during_lying','PSY_Pokal_HRV_SDNN_lying',

'PSY_Pokal_HRV_during_relaxation','PSY_Pokal_HRV_SDNN_ruhend',

'PSY_Pokal_HRV_RMSSD_gesamt','PSY_Pokal_HRV_RMSSD_liegend',

'PSY_Pokal_HRV_RMSSD_ruhend','PSY_Pokal_HRV_LF_gesamt',

'PSY_Pokal_HRV_HF_gesamt','PSY_Pokal_HRV_LF_liegend',

'PSY_Pokal_HRV_HF_liegend','PSY_Pokal_HRV_LF_ruhend',

'PSY_Pokal_HRV_HF_ruhend','PSY_Pokal_HRV_Baevskii_gesamt',

'PSY_Pokal_HRV_Baevskii_liegend','PSY_Pokal_HRV_Baevskii_ruhend',

'PSY_Pokal_HRV_Dauer_unbekannte_Bewegung','PSY_Pokal_HRV_Dauer_liegen',

'PSY_Pokal_HRV_Duration_lying_percentage',

'PSY_Pokal_HRV_Dauer_sitzen_stehen','PSY_Pokal_HRV_Duration_sitting_percentage',

'PSY_Pokal_HRV_Dauer_gehen','PSY_Pokal_HRV_Duration_going_percentage',

'PSY_Pokal_HRV_Dauer_laufen','PSY_Pokal_HRV_Duration_running_percentage','PSY_Pokal_HRV_Dauer_Gurt_nicht_getragen', 'PSY_Pokal_HRV_during_activity', 'PSY_Pokal_HRV_SDNN_during_activity', 'PSY_Pokal_HRV_RMSSD_acitivity', 'PSY_Pokal_HRV_LF_during_activtiy','PSY_Pokal_HRV_HF_during_activity',

'PSY_Pokal_HRV_Baevskii_during_activity'.

With this values no feature engineering was conducted. These were the total measurements by the HRV sensor.

Further no feature engineering was conducted in terms of the laboratory values, the body temperature and blood pressure.

## (S2) Feature importances of base models:

### Biographical Model:

| **Ranking** | **Feature** | **Feature importance** |
| --- | --- | --- |
| **1** | vELSA_Frage_17_Innerhalb_der_letzten_12_Monate_finanzielle_Schwierigkeiten | 0,120 |
| **2** | Haben sie schon mal versucht sich das Leben zu nehmen?_binary | 0,110 |
| **3** | Lubben_2 | 0,108 |
| **4** | Lubben_1 | 0,098 |
| **5** | PSY_Pokal_vELSA_Frage_8_Sport_mind_1_mal_pro_W | 0,089 |
| **6** | Lubben_3 | 0,085 |
| **7** | derzeitiger_Familienstand_rec | 0,083 |
| **8** | Haben sich Ihre Eltern getrennt?_binary | 0,058 |
| **9** | Lubben_5 | 0,043 |
| **10** | Depression_Mutter | 0,040 |
| **11** | Depression_andere | 0,035 |
| **12** | Suicides_close_relatives | 0,031 |
| **13** | Alter_bei_Studieneinschluss | 0,031 |
| **14** | Herzinfarkt unter 60 Jahren_Eltern | 0,022 |
| **15** | addiction_parents | 0,013 |
| **16** | Angst+Zwang_Eltern | 0,013 |
| **17** | Cannabis_n | 0,012 |
| **18** | Arbeitsstunden_Ordinal | 0,009 |

### PHQ15 Model

| **Ranking** | **Feature** | **Feature importance** |
| --- | --- | --- |
| **1** | PSY_Pokal_PHQ-15_15 | 0,401 |
| **2** | PSY_Pokal_PHQ-15_14 | 0,390 |
| **3** | PSY_Pokal_PHQ-15_10 | 0,097 |
| **4** | PSY_Pokal_PHQ-15_8 | 0,061 |
| **5** | PSY_Pokal_PHQ-15_11 | 0,052 |

### Depression Screener Model:

| **Ranking** | **Feature** | **Feature importance** |
| --- | --- | --- |
| **1** | PHQ9_4 | 0,129 |
| **2** | PHQ9_7 | 0,117 |
| **3** | PHQ9_6 | 0,117 |
| **4** | PHQ9_9 | 0,110 |
| **5** | WHO5_1 | 0,109 |
| **6** | PHQ9_2 | 0,106 |
| **7** | PHQ9_3 | 0,099 |
| **8** | WHO5_5 | 0,079 |
| **9** | PHQ9_1 | 0,056 |
| **10** | PHQ9_5 | 0,039 |
| **11** | WHO5_3 | 0,038 |

### All clinical model:

| **Ranking** | **Feature** | **Feature importance** |
| --- | --- | --- |
| **1** | PHQ9_4 | 0,112 |
| **2** | PSY_Pokal_WHODAS2.0_11_clipped | 0,078 |
| **3** | PSY_Pokal_WHODAS2.0_5_clipped | 0,074 |
| **4** | PHQ9_6 | 0,069 |
| **5** | PHQ9_7 | 0,067 |
| **6** | PSY_Pokal_WHODAS2.0_4_clipped | 0,065 |
| **7** | PSY_Pokal_WHODAS2.0_12_clipped | 0,064 |
| **8** | PSY_Pokal_WHODAS2.0_6_clipped | 0,063 |
| **9** | WHO5_1 | 0,059 |
| **10** | PHQ9_2 | 0,057 |
| **11** | PHQ9_3 | 0,055 |
| **12** | PHQ9_9 | 0,054 |
| **13** | WHO5_5 | 0,046 |
| **14** | PSY_Pokal_WHODAS2.0_10_clipped | 0,038 |
| **15** | PHQ9_1 | 0,032 |
| **16** | PSY_Pokal_WHODAS2.0_2_clipped | 0,026 |
| **17** | PHQ9_5 | 0,023 |
| **18** | WHO5_3 | 0,012 |
| **19** | UCLA3 | 0,008 |

### Allself rated model

| **Ranking** | **Feature** | **Feature importance** |
| --- | --- | --- |
| **1** | PSY_Pokal_WHODAS2.0_5_clipped | 0,098 |
| **2** | PHQ9_4 | 0,086 |
| **3** | PSY_Pokal_WHODAS2.0_11_clipped | 0,073 |
| **4** | WHO5_5 | 0,069 |
| **5** | PSY_Pokal_WHODAS2.0_6_clipped | 0,066 |
| **6** | PSY_Pokal_WHODAS2.0_12_clipped | 0,065 |
| **7** | PHQ9_9 | 0,063 |
| **8** | PHQ9_1 | 0,063 |
| **9** | PHQ9_7 | 0,063 |
| **10** | PHQ9_2 | 0,062 |
| **11** | PHQ9_6 | 0,061 |
| **12** | PSY_Pokal_WHODAS2.0_4_clipped | 0,061 |
| **13** | WHO5_1 | 0,059 |
| **14** | PSY_Pokal_WHODAS2.0_10_clipped | 0,049 |
| **15** | PHQ9_3 | 0,040 |
| **16** | WHO5_3 | 0,022 |

## (S3) Performance of the Stacked models

Various stacked models combining different base models were tested. No stacked models achieved higher BAC compared to the best performing base model used by them, as a result, no stacked model is needed for the clinical application.

| **Model name** | **Model type** | **Modalities** | **Features** | **Pipeline** | **BAC** | **Sens** | **Spec** |
| --- | --- | --- | --- | --- | --- | --- | --- |
| **Biograph + Somatic** | Stacked | Prediction scores | 2 | P1 | 78,3 | 75,6 | 81,1 |
| **Clinical 5 + Biograph** | Stacked | Prediction scores | 2 | P1 | 84,9 | 81,1 | 88,6 |
| **Clinical 5 + Somatic** | Stacked | Prediction scores | 2 | P1 | 85,0 | 82,2 | 87,8 |
| **Clinical 5 + Biograph + Somatic** | Stacked | Prediction scores | 3 | P1 | 85,4 | 83,3 | 87,4 |
| **Clinical 10 + Biograph** | Stacked | Prediction scores | 2 | P1 | 83,2 | 77,8 | 88,6 |
| **Clinical 10 + Somatic** | Stacked | Prediction scores | 2 | P1 | 84,9 | 81,1 | 88,6 |
| **Clinical 10 + Biograph + Somatic** | Stacked | Prediction scores | 3 | P1 | 85,7 | 83,3 | 88,0 |
| **Clinical 15 + Biograph** | Stacked | Prediction scores | 2 | P1 | 86,7 | 85,6 | 87,8 |
| **Clinical 15 + Somatic** | Stacked | Prediction scores | 2 | P1 | 86,0 | 86,7 | 85,3 |
| **Clinical 15 + Biograph + Somatic** | **Stacked** | **Prediction scores** | **3** | **P1** | **87,8** | **90,0** | **85,5** |

## (S4) Configuration details

### XGBoost hyper-parameters in terms of the *Clinical 15* algorithm

params = {

'booster': ['gbtree'],

'objective': ['binary:logistic'],

'learning_rate':[0.01,0.001],

'n_estimators':[500,1000,2000],

'subsample': [0.6,0.9],

'colsample_bytree': [0.12,0.24,0.36],

'learning_rate': [0.01],

'max_depth': [1,3],

'gamma':[3,5,7],

'seed': [2],

'silent': [1],

‘scale_pos_weight’:[Counter(train[target2].values)[0]/Counter(train[target2].values)[1]]

}

| **Colour** | **Prediction** | **Prediction confidence** |
| --- | --- | --- |
| **Red** | Positive | High / Medium |
| **Yellow** | Positive / Negative | Low |
| **Green** | Negative | High / Medium |

## (S5) Variables used to establish GMM model

somatic_params=[ 'Alter_bei_Studieneinschluss',

'SEX',

'PSY_Pokal_Waist_to_hip_ratio',

'PSY_Pokal_Bauchumfang',

'PSY_Pokal_Hueftumfang',

'PSY_Pokal_Parameter_Puls',

'PSY_Pokal_Diastolischer_Blutdruck:',

'PSY_Pokal_Systolischer_Blutdruck:',

'PSY_Pokal_Messung_Koerpertemperatur',

'PSY_Pokal_HRV_during_lying',

'PSY_Pokal_HRV_SDNN_lying',

'PSY_Pokal_HRV_RMSSD_liegend',

'PSY_Pokal_HRV_LF_liegend',

'PSY_Pokal_HRV_HF_liegend',

'PSY_Pokal_HRV_Baevskii_liegend',

'Wert_Glucose',

'Wert_CRP',

'Wert_GOT',

'Wert_GPT',

'Wert_Gamma-GT',

'Wert_Cholesterin',

'Wert_Triglyzeride',

'Wert_LDL-Cholesterin',

'Wert_HDL-Cholesterin',

'Wert_non-HDL-Cholesterin',

'Wert_HbA1c_NGSP ',

'Wert_Leukozyten',

'Wert_Erythrozyten',

'Wert_Hämoglobin',

'Wert_Neutrophile_Granulozyten',

'Wert_Neutrophile_Granulozyten_G',

'Wert_Eosinophile_Granulozyten',

'Wert_Hämatokrit ',

'Wert_MCV ',

'Wert_MCH',

'Wert_MCHC',

'Wert_Thrombozyten',

'Wert_RDW-CV-corr',

'Wert_PDW',

'Wert_MPV ',

'Wert_Monozyten',

'Wert_Eosinophile_Granulozyten_Percent',

'Wert_Basophile_Granulozyten_Percent',

'Wert_Lymphozyten_G',

'Wert_Monozyten_G',

'Wert_Basophile_Granulozyten_G ',

'Wert_Alpha-1-Antitrypsin[g/l]',

'TSH',

'Cortisol ',

'IGF-1 ']

## (S6 ) QQ-Plot


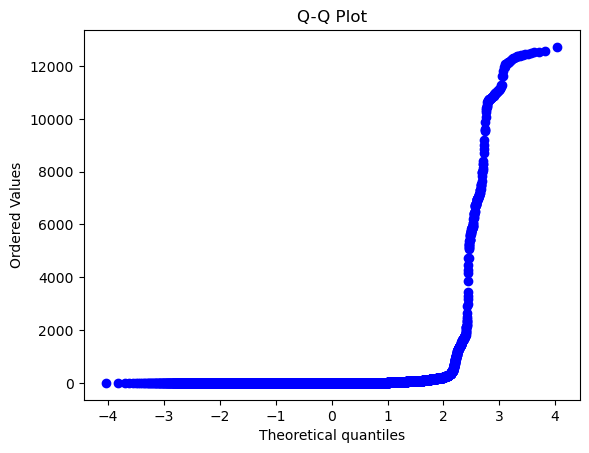


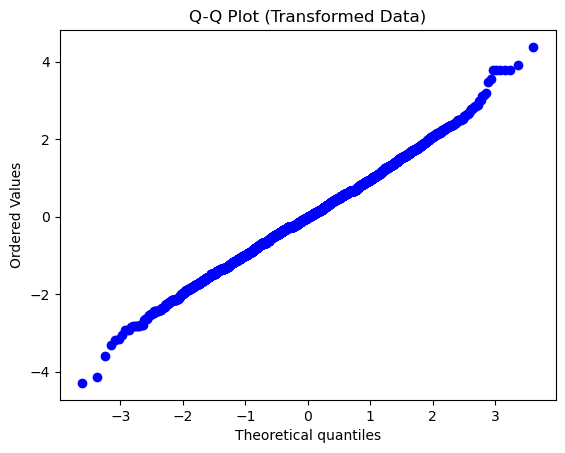


## (S7 ) Metrices of different GMM configurations


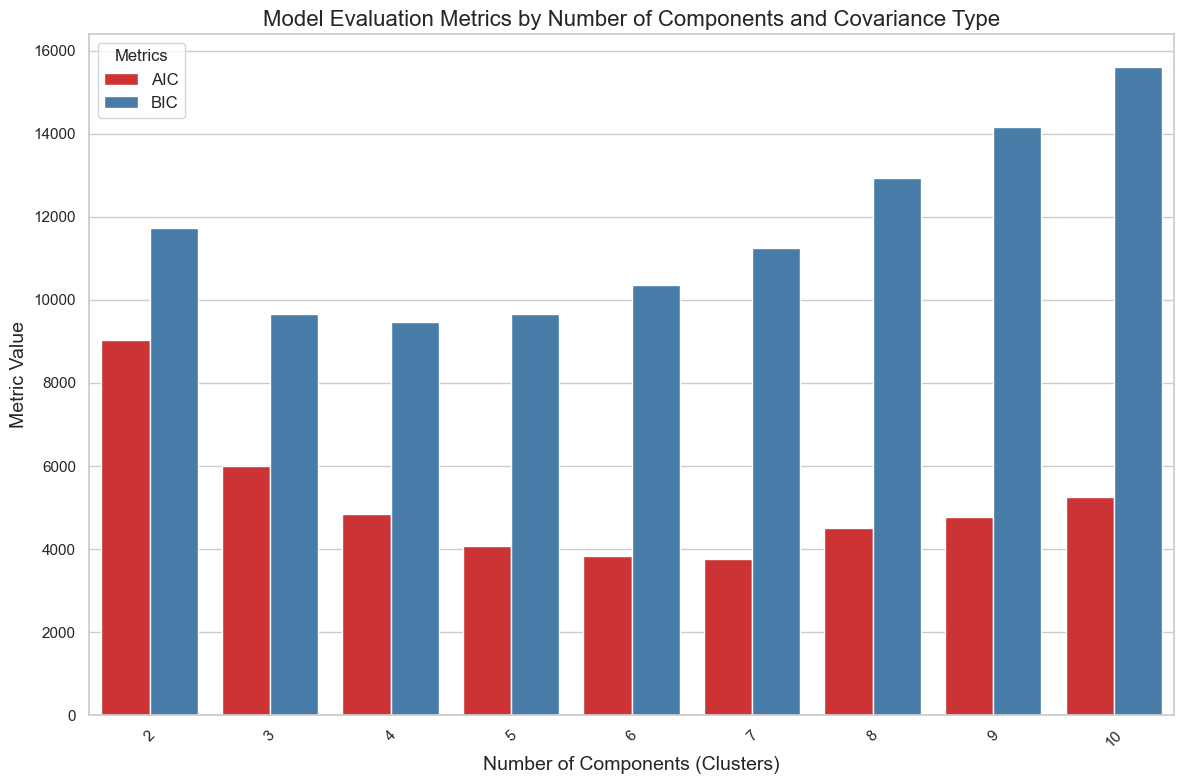


|  | **n_components** | **covariance_type** | **AIC** | **BIC** | **Silhouette Score** |
| --- | --- | --- | --- | --- | --- |
| **0** | 2 | full | 3671.536757 | 10298.532193 | 0.097974 |
| **1** | 3 | full | -8352.098084 | 1589.644974 | 0.062957 |
| **2** | 4 | full | -12578.104380 | 678.386302 | 0.066797 |
| **3** | 5 | full | -15786.385449 | 784.852856 | 0.058749 |
| **4** | 6 | full | -16334.683561 | 3551.302367 | 0.052036 |
| **5** | 7 | full | -16436.833942 | 6763.899609 | 0.052680 |
| **6** | 8 | full | -12148.477351 | 14367.003822 | 0.057703 |
| **7** | 9 | full | -9880.580284 | 19949.648512 | 0.052891 |
| **8** | 10 | full | -7964.800037 | 25180.176382 | 0.049045 |
| **9** | 2 | tied | 8187.002424 | 11626.740530 | 0.097974 |
| **10** | 3 | tied | 8179.914044 | 11747.142443 | 0.060464 |
| **11** | 4 | tied | 8219.804228 | 11914.522921 | 0.066797 |
| **12** | 5 | tied | 8224.859469 | 12047.068455 | 0.057089 |
| **13** | 6 | tied | 8243.226634 | 12192.925913 | 0.052467 |
| **14** | 7 | tied | 8288.563296 | 12365.752868 | 0.052680 |
| **15** | 8 | tied | 8179.005586 | 12383.685451 | 0.057703 |
| **16** | 9 | tied | 8210.877688 | 12543.047847 | 0.052891 |
| **17** | 10 | tied | 8205.923407 | 12665.583859 | 0.049045 |
| **18** | 2 | diag | 11735.102174 | 12237.563918 | 0.099176 |
| **19** | 3 | diag | 11730.054607 | 12484.997128 | 0.070747 |
| **20** | 4 | diag | 11475.780532 | 12483.203829 | 0.045937 |
| **21** | 5 | diag | 11619.751460 | 12879.655534 | 0.052969 |
| **22** | 6 | diag | 11143.471100 | 12655.855950 | 0.049396 |
| **23** | 7 | diag | 10936.269575 | 12701.135202 | 0.046333 |
| **24** | 8 | diag | 10336.586460 | 12353.932864 | 0.038031 |
| **25** | 9 | diag | 9802.275309 | 12072.102489 | 0.034238 |
| **26** | 10 | diag | 9875.682129 | 12397.990086 | 0.038763 |
| **27** | 2 | spherical | 12517.106940 | 12774.587336 | 0.100198 |
| **28** | 3 | spherical | 12429.627005 | 12817.097504 | 0.052277 |
| **29** | 4 | spherical | 12305.576411 | 12823.037012 | 0.062684 |
| **30** | 5 | spherical | 12250.918903 | 12898.369608 | 0.057198 |
| **31** | 6 | spherical | 12260.814442 | 13038.255249 | 0.050788 |
| **32** | 7 | spherical | 12241.498559 | 13148.929470 | 0.052385 |
| **33** | 8 | spherical | 11622.860188 | 12660.281201 | 0.056642 |
| **34** | 9 | spherical | 10941.825853 | 12109.236969 | 0.053905 |
| **35** | 10 | spherical | 10900.416030 | 12197.817249 | 0.049672 |
